# The diversity of resident passerine bird in the East Yunnan‐Kweichow Plateau is closely related to plant species richness, vertical altitude difference and habitat area

**DOI:** 10.1002/ece3.9735

**Published:** 2023-01-17

**Authors:** Haibo Zhang, Lingbin Yan, Lifei Yu, Haijun Su, Canshi Hu, Mingming Zhang, Zhihong Kong

**Affiliations:** ^1^ College of Life Sciences, Guizhou University Guiyang China; ^2^ The Key Laboratory of Plant Resources Conservation and Germplasm Innovation in Mountainous Region (Ministry of Education) & Collaborative Innovation Center for Mountain Ecology and Agro‐Bioengineering (CICEAB), Institute of Agro‐Bioengineering & College of Life Sciences Guizhou University Guiyang China; ^3^ Aha Lake National Wetland Park Guiyang China; ^4^ Forestry College, Guizhou University Guiyang China; ^5^ Research Center for Biodiversity and Natural Conservation Guizhou University Guiyang China

**Keywords:** altitude, area, bird, East Yunnan‐Kweichow Plateau, functional structure, phylogenetic structure, plant species richness

## Abstract

East Yunnan‐Kweichow Plateau is rich in biodiversity in China. Complex geographical and climatic conditions and rich bird resources made this area an ideal system for studying the spatial distribution mechanism and influencing factors of birds, which were still unknown. Bird community data from 37 sites in this region were collected, including 505 bird species and 164 species of resident passerine bird, were extracted for analysis. The taxonomic diversity, phylogenetic diversity, functional diversity (FD), and community structure index were calculated. Ordinary least square (OLS), spatial autoregressive models (SAR), and structural equation model (SEM) were used to explore the relationship between bird diversity index and environmental factors which were used to describe the habitat conditions of birds. Results indicated that species richness (SR), phylogenetic diversity (PD), and FD have similar distribution patterns and are mainly affected by vascular plant species richness, habitat area, and vertical altitude difference. The phylogenetic and functional structure of bird community changed in both longitude and latitude direction, and the phylogenetic structure of community was dispersed in the west and clustered in the east, significantly related to habitat area, and vertical altitude difference, the functional structure was dispersed in all sites, significantly related to average annual precipitation. The community in the west and south of the East Yunnan‐Kweichow Plateau is mainly driven by interspecific competitive, while the process in the east and north is mainly driven by environmental filtration. Distribution pattern of bird diversity was caused by the comprehensive action of various habitat factors which were mainly related to food availability and habitat heterogeneity. For maintaining the living space of birds, the protection of urban parks, wetland parks, campuses, and other urban green spaces is as important as national and provincial nature reserves. Revealing the construction mechanism and main influencing factors of bird communities in different areas is conducive to targeted protection work.

## Graphical abstract

The bird diversity in the East Yunnan‐Kweichow Plateau was analyzed on a large spatial scale for the first time. The distribution pattern and main influencing factors of bird diversity in East Yunnan‐Kweichow Plateau were analyzed from three dimensions: taxonomic diversity, phylogenetic diversity, and functional diversity. This paper preliminarily revealed that the main driving factors of the spatial distribution of bird diversity in the East Yunnan‐Kweichow Plateau were plant species richness, vertical elevation difference, and habitat area. Structural equation model (SEM) was used for data analysis in this paper.

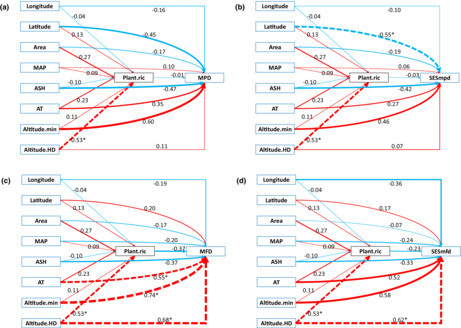

## 1 INTRODUCTION

Exploring the ecological mechanism and driving factors of large‐scale geographical distribution differences of species communities is not only a basic problem in ecological research but also one of the hotspots in biogeography research (Currie, 1991; MacArthur, 1984). The distribution pattern of community diversity was affected by many factors, such as habitat heterogeneity caused by vertical altitude difference (Novillo & Ojeda, 2014; Walter & Carsten, 2002), food availability and vegetation structure (Ferger et al., 2014; Zhang et al., 2013), regional energy and precipitation (Graham et al., 2009; Mccain, 2007, 2009), and historical factors, such as historical geological events or climatic events (Fjeldsaå & Lovett, 1997; Qu et al., 2014). Among them, vegetation and climate are the two most important factors that affect the structure of bird community (Renner & Bates, 2020).

The number of species in a community, known as taxonomic diversity, is commonly used to characterize biodiversity and species assemblages (He et al., 2019; Pan et al., 2016), but taxonomic diversity alone ignores other axes of variation that exist within a community, such as variation in ecology (functional diversity (FD)) and the degree of relatedness among species (phylogenetic diversity (PD); Teja Tscharntke et al., 2012). The early research on community assemblages mainly regarded all species as ecological equivalents of independent evolution from the point of view of the change of species diversity, which could not explain the evolution history and functional characteristics of species, and could not accurately reveal the causes of community construction (Swenson et al., 2012; Webb et al., 2002). In the past decade, ecologists have chosen FD and PD to explore the mechanism of community assemblage (Che et al., 2019; Swenson, 2013; Swenson et al., 2012; Webb et al., 2002). FD, which can be calculated from species' trait data, reflects the scope of functional traits within an assemblage, providing insight into the ecological uniqueness, redundancy, or complementarity within a community. Since the early 1990s, a large number of groundbreaking papers on FD have been published (Naeem et al., 1994; Tilman & Downing, 1994). The functional role of species in the community is determined by functional traits (McGilla et al., 2006), such as the types of habitats they distribute and the way they interact (Davies et al., 2007; Dethier, 1994). FD indicates the coexistence of species by the diversity of functional traits (Mouchet et al., 2010). The higher the FD of the community, the stronger the divergence of species traits, and the species coexist stably because of the high differentiation of niche in the community caused by the high difference of survival strategies (Spasojevic & Suding, 2012). PD reflects the phylogenetic variation among species and the evolutionary history of a community (Faith, 1992; Hanz et al., 2019) and focuses on studying the species composition of the community from the perspective of evolution (Jia et al., 2020), reflecting the pattern of species coexistence (Arroyo‐Rodríguez et al., 2012; Cavender‐Bares et al., 2009; Si et al., 2017). Compared with traditional taxonomic diversity, PD and FD can provide more information, especially in the process of community construction (Cavender‐Bares et al., 2009; Meynard et al., 2011), better reflect the evolutionary characteristics and ecological differences of bird communities (Barbaro et al., 2013; Cadotte et al., 2011, 2019; Liang et al., 2020; Meynard et al., 2011; Petchey & Gaston, 2010; Sandel, 2018; Si et al., 2016; Zhang et al., 2020). Therefore, they have more obvious advantages in mastering the mechanism of community assembly processes (Hanz et al., 2019; McGilla et al., 2006), habitat filtration leads to a clustered community (Petchey et al., 2007), and interspecific competition leads to a divergent community (Ackerly et al., 2006). Therefore, integrating the three complementary dimensions of taxonomic diversity, PD, and FD to describe the spatio‐temporal distribution pattern of the community has become an important method to reveal the mechanism of assemblages (Cadotte et al., 2013; He et al., 2018; Huang et al., 2012; Monnet et al., 2014; Swenson, 2011; Webb et al., 2002). Whether community construction is driven by environmental filtering (Gleason, 1926), biotic interactions (Clements, 1916), or dispersal processes (Hubbell, 2001) alone, it has been controversial for a long time (Ellwood et al., 2009; Pavoine & Bonsall, 2011). In recent years, more studies on the integration of PD or functional traits show that, these mechanisms may occur at the same time (Helmus et al., 2007) or appear along the environmental gradient (Mason et al., 2007). Therefore, the key problem at present is to determine which mechanism plays a major role in community construction, rather than which mechanism drives the community construction process (Mouchet et al., 2010).

Birds are sensitive to habitat changes, the functional roles of birds can be described by morphological or life history traits related to their food preference, flying ability, and exercise habits and has become an ideal object for the study of community distribution differences and ecological mechanisms (Luck et al., 2012; Pigot et al., 2016); meanwhile, the functional traits and phylogenetic relationships of most known birds in the world are relatively clear (International, B, 2022; Jetz et al., 2012; Wang et al., 2021). Passeriformes is a large community coexisting in the forest and an ideal group to study the mechanism of species symbiosis and community construction (He et al., 2018).

Yunnan‐Kweichow Plateau is located in the second ladder of China, including the eastern part of Yunnan Province, the whole Kweichow province, the northwest of Guangxi and the borders of Sichuan province, Hubei province and Hunan province, and the area is about 180,000 km^2^. This area was characterized by typical karst geomorphology, and its elevation ranges from 137 to 2900 m. Climate in this area is diverse which belongs to the subtropical warm and humid monsoon climate zone, affected by atmospheric circulation and topography. The mean annual temperature is about 15°C. The annual precipitation in this region is unevenly distributed, with the average annual precipitation ranging from 1100 to 1300 mm. The average annual sunshine hours range from 12:00 to 15:00 h. The spatial distribution of vegetation in this area is obviously transitional, various vegetation types overlap and intricate in geographical distribution. The unique natural geographical conditions and climatic characteristics have given birth to a wealth of wild animals and plants in this area. There are 505 species of birds counted in this study. Complex and diverse geographical, climatic conditions, and rich bird resources have made this area an ideal system for studying the geographical distribution pattern of bird diversity, while the bird diversity distribution patterns, the main ecological processes, and the key influencing factors of community construction are still unknown.

General survey data of birds from 37 sites in this area were collected, and the data of resident passerine bird were extracted. Combining with bird functional traits and phylogenetic data, the species richness, PD and FD of birds and their relationship with environmental factors were analyzed, and tried to test: (1) the distribution pattern of taxonomic diversity, PD and FD of birds on a larger spatial scale. (2) What is the assembly mechanism of bird communities at different sites? (3) What are the main habitat factors driving bird diversity and community assembly? By jointly analyzing various metrics of diversity and environmental factors, we hope to better understand how biotic and abiotic factors interact to shape avian communities in this area.

## 2 MATERIALS AND METHODS

### 2.1 Bird distribution data

By searching for keywords such as “Yunnan‐Kweichow Plateau,” “Guizhou/Kweichow,” “bird,” “scientific expedition” and “nature reserve” on Web of Science, Google Scholar, CNKI, Wanfang Data and other literature platforms, we downloaded relevant literatures, and we collected monographs published or to be published in this region (Appendix S1), bird community data from 37 sites were collected and analyzed, including 31 nature reserves, four parks, and two campuses (Appendix S2). To further ensure the adequate sampling intensity of the survey, we only selected bird list that has been investigated for at least 1 year to create a 0–1 matrix of bird species at each location. Only resident passerine bird was chosen for analysis to reduce the deviation caused by seasonal and long‐distance migratory birds in the survey data (Mccain, 2009).

### 2.2 Phylogenetic trees

In order to analyze the PD of bird community, we used 164 species of resident passerine bird species to construct a bird phylogenetic tree based on the global bird phylogenetic system (http://www.birdtree.org), and downloaded 5000 randomly distributed phylogenetic trees from Hackett All Species: a set of 10,000 trees with 9993 OTUS each. TreeAnnonator (v1.10.4) in BEAST package was used to construct a phylogenetic tree with the maximum confidence value of average height, and then Figtree (v1.4.4) software was used to output the phylogenetic tree, based on which the PD was analyzed (Appendix S4).

### 2.3 Phylogenetic signal

More and more studies on functional traits analyze the synergy or coupling relationship between traits and their environmental adaptability under the background of interspecies phylogeny (evolutionary history; Ackerly et al., 2006; Letcher et al., 2015; Pavoine et al., 2010; Yang et al., 2014). The phylogenetic niche conservatism hypothesis holds that closely related species should have higher similarity in functional traits and their niches are more similar than those of unrelated species (Losos, 2010). When analyzing the correlation of functional traits in local communities or on a larger scale, testing the phylogenetic signals of functional traits in advance is often necessary. Phylogenetic community structure is greatest when traits are highly conserved and when multiple traits influence species membership in communities (Kraft et al., 2007), and if functional traits are phylogenetic conservative, the coupling relationship between functional traits should be analyzed under the background of considering the phylogenetic information among species (Garland Jr et al., 1992). Therefore, for continuous traits, Blomberg's K was used to detect the phylogenetic signals (Blomberg Jr et al., 2003; Appendix S7), A value of *K* ≥ 1 suggests a significant phylogenetic signal in trait data that departs from a model of trait evolution under Brownian motion (i.e. trait conservatism), while a value of *K* < 1 suggests less trait conservatism than expected under a model of Brownian motion (i.e. trait convergence). For binary traits, *D*‐statistic was used to evaluate phylogenetic signals (Fritz & Purvis, 2010). If *D* approaches 0, then there is conserved trait evolution. *D* < 0 indicates that the binary trait under Brownian motion is more conservative than expected, a value of *D* ≥ 1 indicates no phylogenetic signal or that a trait is over‐dispersed on the phylogenetic tree. Blomberg's *K* and *D*‐statistic values are calculated using the “phytol” and “caper” in R packages, respectively.

### 2.4 Functional traits of birds

To evaluate the FD of bird community, nine functional traits which can reflect the functional role of birds in natural ecosystem were selected for the construction of functional traits cluster tree and FD analysis (Appendix S5). The nine traits include the following: seven continuous traits (body mass, wing length, culmen, tarsus length, clutch size, generation length) and three categorical traits (eight binary diet traits, six binary foraging stratum traits, six binary nest location traits). Bird morphological traits and clutch size were derived from a recently published dataset (Wang et al., 2021), which collects 17 functional character data of 1445 bird species in China. The data of diet, foraging stratum and nest location of birds were extracted from the fauna of China (Zhao, 2001), and the generation length was obtained from the official websites of Birdlife and IUCN.

### 2.5 Environmental data

Habitat types and vegetation diversification can provide a variety of nutritional niches for birds, such as food resources and foraging layer (Strohbach et al., 2015), and create microhabitats for birds foraging and nesting (Ahnström et al., 2008), so as to maintain the level of bird diversity. Considering the importance of environmental factors in determining the distribution of bird diversity, we choose 12 potential factors related to bird distribution pattern to describe the habitat characteristics of bird species (Appendix S6), which were related to spatial geographical location, habitat size, availability of food resources, climate, and altitude. Spatial geographical factors include longitude and latitude, habitat size was indicated by habitat area (Area), food resource availability was indicated by vascular plant species richness (Plant.ric), climatic factors include mean annual temperature (MAT), mean annual precipitation (MAP), annual sunshine time (AST), accumulated temperature (AT), and altitude factors include mean altitude (Altitude.mean), the lowest altitude (Altitude.min), the highest altitude (Altitude.max) and vertical altitude difference (Altitude.HD). Habitat factor data mainly come from local comprehensive scientific investigation monographs, which are obtained by experts from different disciplines through field investigation, analysis and summary, and are scientific and accurate, a small number of missing factor data are supplemented by the local government official website and literature (Appendix S1).

### 2.6 Statistical analysis

We calculated taxonomic diversity, FD, and PD of birds in 37 sites. Taxonomic diversity was represented by the number of resident passerine bird species (SR). PD was represented by Faith' PD index (PD), PD is the smallest sum of branch length of all species in a local community, and the branch length represents the evolution time. The higher the PD value, the longer the evolutionary time that a community accumulate. PD was implemented in the R package “picante”. The calculation of FD is similar as PD. Because the functional trait indices involve continuous and binary variables, the functional trait matrix was transformed into a similarity matrix by using Gower‐distance (Gower, 1966), which can deal with these two data types at the same time, that is, the functional distance between all species was quantified by giving the same weight to different types of variables. Then, the unweighted pair‐group method with arithmetic mean (UPGMA) was used to cluster the similarity matrix to establish a functional clustering tree, based on which the FD was calculated.

In order to evaluate the phylogenetic structure of bird community, the mean pairwise phylogenetic distance (MPD) and the standardized effect size of MPD (SESmpd) were calculated.

$$\text{SESmpd}=-1\times \left({\mathrm{MPD}}_{\mathrm{obs}}-{\text{meanMPD}}_{\mathrm{rnd}}\right)/{\text{sdMPD}}_{\mathrm{rnd}}$$

MPD_obs_ is the observed mean pairwise phylogenetic distance of birds in a site, meanMPD_rnd_ the metric corresponding mean value of the 999 randomly simulated communities, and sdMPD_rnd_ is the standard deviation of the null model.

In order to evaluate the functional structure of bird community, the mean pairwise functional distance (MFD) and its standardized effect size (SESmfd) were calculated.

$$\text{SESmfd}=-1\times \left({\mathrm{MFD}}_{\mathrm{obs}}-{\text{meanMFD}}_{\mathrm{rnd}}\right)/{\text{sdMFD}}_{\mathrm{rnd}}$$

MFD_obs_ is the observed value of the mean pairwise functional distance of birds in a site, meanMFD_rnd_ is the metric corresponding mean value of the 999 randomly simulated communities, and sdMFD_rnd_ is the standard deviation of the null model.

The SES index <0 indicates that the species in the community are closer than the expected, that is, the community is clustered; on the contrary, the SES value >0 indicates that the species in the community is over‐dispersed than the expected, if SES is equal to 0, it means that the phylogeny or FD of the community is no different from the null model, and the community shows a random pattern. According to the prediction of the conservative hypothesis of phylogenetic niche (Losos, 2010), the clustered community phylogenetic structure indicates that environmental filtration plays a leading role in the community construction, while the dispersed community phylogenetic structure is driven by competitive exclusion or mutually beneficial symbiosis.

In order to test the correlation between diversity indices (SR, PD, FD) and environmental factors separately, ordinary least square (OLS) regression was used. To make the regression coefficients comparable, all variables were standardized (mean is 0, standard deviation is 1). OLS analysis was done by using the “car” in R packages.

Spatial autocorrelation is a frequent phenomenon in ecological data and can affect estimates of model coefficients and inference from statistical models (Daniel Kissling & Carl, 2008). The presence of spatial autocorrelation violates the assumption of independently distributed errors in regression models, and, as a consequence, Type I errors of traditional tests might be inflated (Legendre, 1993). Moreover, spatial autocorrelation can affect inference from statistical models and our ability to evaluate the importance of explanatory variables (Diniz‐Filho et al., 2003; Dormann et al., 2007). To explore the influence of spatial autocorrelation on inference from our path models, we tested for the presence of spatial autocorrelation by calculating Moran's *I* values (i.e., a measure of spatial autocorrelation; Legendre, 1993) on the residuals of our minimal adequate (OLS) regression models. Because of the significant spatial autocorrelation in residuals, we fitted Spatial autoregressive models (SAR), which can include the spatial autocorrelation structure of a given data set. Final model assessment was based on the reduction of spatial autocorrelation in model residuals (evaluated with Moran's *I* values). To compare the relative importance of predictor variables from SAR and OLS regressions, we calculated standardized partial regression coefficients from both model types. Moran's I values and SAR were calculated using the R library ‘spdep’.

The structural equation model (SEM) was used to further verify the direct or indirect effects of habitat factors on bird diversity and community structure. Considering the low requirements for the normality and independence of sample size and dependent variable variance, Piecewise SEM was used. In order to avoid the influence of multicollinearity of environmental variables (Appendix S11), only nine factors were included in SEM analysis. SEM was completed by the “piecewise SEM” in R packages.

## 3 RESULTS

There 505 species of birds obtained in 37 sites in the east Yunnan‐Kweichow Plateau, of which 164 species of passerine resident birds were used for analysis in this paper. In this study, phylogenetic signals were significantly related to continuous traits and most categorical traits, indicating strong phylogenetic niche conservatism (Appendix S7). Specifically, all continuous traits have significant phylogenetic signals (*p* < .05), with wing length having the strongest signal (*K* = 1.29). For binary traits, *D*‐statistic analysis showed that 11 binary traits had significant phylogenetic signals (*D* < 0 and *p* < .05), while the other binary traits had relatively weak phylogenetic signals.

**FIGURE 1:**
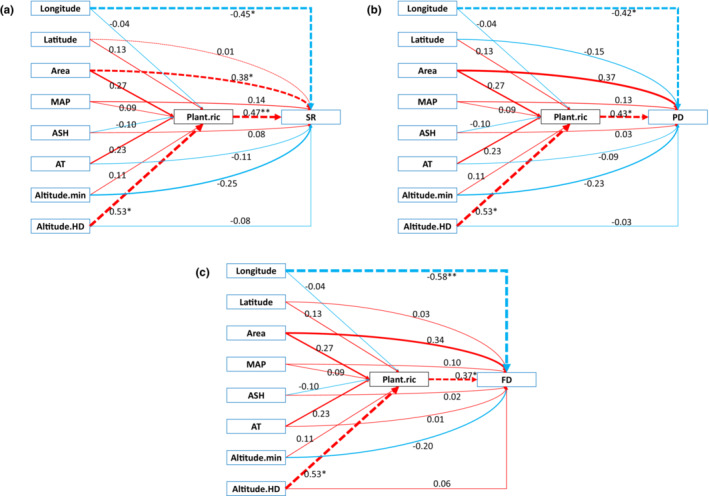
Results of structural equation model (SEM) examining the direct and indirect effects of longitude, latitude, area, mean annual precipitation (MAP), annual sunshine hours (ASH), accumulated temperature (AT), the lowest altitude (Altitude.min), vertical altitude difference (Altitude.HD), and plant species richness (Plant.ric) on resident passerine bird species richness (SR, a), phylogenetic diversity (PD, b), and functional diversity (FD, c). Red represents a positive relationship and green represents a negative relationship. Dashed lines indicate significant correlations. Standardized regression coefficients were given, and the strength of the effects was proportional to line width. *p < .05, **p < .01.

Bird species richness (SR), PD and FD have similar distribution patterns (Table 1, Figures 1 and 3), which are higher in the north, south and west, such as Fanjing Mountain (FJS, SR = 91, FD = 4.28, PD = 1683.47), Dashahe (DSH SR = 90, FD = 4.45, PD = 1624.86), Maolan (ML SR = 87, FD = 4.17, PD = 1729.52), and lower in the middle and east like parks and campuses with lower protection levels.

**TABLE 1:** Relationships between resident passerine bird taxonomic diversity, phylogenetic diversity, functional diversity and each associated variable by ordinary least squares (OLS), spatial autoregressive models (SAR), and Moran test for the model residuals.

	Longitude	Latitude	Area	Plant.ric	MAT	MAP	ASH	AT	Altitude.min	Altitude.max	Altitude.mean	Altitude.HD
**Taxonomic diversity**												
SR												
OLS												
Coef_ols	−3.29	0.92	0.27	0.37	−0.26	0.32	−0.06	−0.44	−0.13	0.55	0.20	0.42
r2_ols	0.02	0.02	0.37**	0.46**	0.02	0.03	0.00	0.09	0.09	0.24**	0.04	0.40**
*I_ols*	0.21	0.16	0.19	0.32*	0.22	0.22	0.20	0.14	0.19	0.20	0.21	0.09
SAR												
Coef_sar	0.14	−0.13	0.60	0.67	−0.15	0.19	−0.04	−0.28	−0.21	0.48	0.21	0.62
AIC	110.28	110.46	93.82	88.33	110.25	109.64	111.01	107.85	109.39	100.81	109.41	92.28
*I_sar*	−0.01	−0.01	0.02	0.12	0.01	0.01	−0.01	−0.04	−0.01	0.02	0.02	−0.06
**Phylogenetic diversity**												
PD												
OLS												
Coef_ols	−2.49	0.00	0.19	0.27	−0.13	0.32	0.02	−0.27	−0.10	0.40	0.14	0.32
r2_ols	0.02	0.00	0.32**	0.39**	0.01	0.06	0.00	0.00	0.05	0.21**	0.04	0.37**
*I_ols*	0.20	0.16	0.21	0.32*	0.21	0.20	0.19	0.17	0.21	0.19	0.21	0.13
SAR												
Coef_sar	0.02	−0.12	0.56	0.62	−0.09	0.23	0.00	−0.22	−0.22	0.45	0.19	0.60
AIC	111.06	110.50	96.64	92.28	110.74	108.91	111.08	109.13	109.17	102.26	109.75	93.76
*I_sar*	−0.01	−0.01	0.02	0.10	0.00	0.00	−0.01	−0.03	−0.01	0.01	0.01	−0.06
MPD												
OLS												
Coef_ols	−0.37	−0.20	−0.01	−0.01	0.01	0.01	0.03	0.02	0.01	−0.01	0.01	−0.02
r2_ols	0.06	0.21**	0.22**	0.10	0.00	0.01	0.05	0.03	0.12*	0.01	0.02	0.14*
*I_ols*	−0.27	−0.09	−0.12	−0.09	−0.02	−0.08	−0.20	−0.04	−0.14	−0.03	−0.08	−0.17
SAR												
Coef_sar	−0.52	0.00	−0.47	−0.32	0.04	0.12	0.26	0.16	0.39	−0.10	0.17	−0.39
AIC	101.79	109.35	102.52	107.94	111.90	111.41	109.60	110.99	106.36	111.61	110.93	105.94
*I_sar*	0.00	0.02	−0.05	0.02	0.01	−0.02	−0.07	−0.02	0.05	0.00	0.00	−0.03
SESmpd												
OLS												
Coef_ols	−16.68	−12.98	−0.79	−0.71	1.00	0.75	1.56	1.23	0.65	−0.70	0.25	−0.98
r2_ols	0.05	0.30**	0.26**	0.13*	0.02	0.02	0.07	0.05	0.09	0.03	0.01	0.17*
*I_ols*	−0.39	−0.07	−0.10	−0.09	0.01	−0.09	−0.24	−0.07	−0.09	−0.04	−0.06	−0.16
SAR												
Coef_sar	−0.64	−0.22	−0.51	−0.38	0.15	0.13	0.30	0.23	0.33	−0.18	0.08	−0.43
AIC	95.52	110.14	100.97	106.38	111.17	111.33	108.77	109.99	107.93	110.78	111.71	104.70
*I_sar*	0.00	0.01	−0.05	0.03	0.03	−0.03	−0.09	−0.03	0.04	0.00	0.00	−0.03
**Functional diversity**												
FD												
OLS												
Coef_ols	−3.77	0.58	0.16	0.23	−0.18	0.13	−0.01	−0.28	−0.07	0.38	0.16	0.28
r2_ols	0.06	0.02	0.29**	0.36**	0.02	0.01	0.00	0.07	0.03	0.25**	0.06	0.38**
*I_ols*	0.26	0.16	0.29	0.37*	0.25	0.24	0.24	0.16	0.26	0.16	0.22	0.15
SAR												
Coef_sar	0.14	−0.21	0.52	0.59	−0.14	0.11	−0.03	−0.25	−0.18	0.48	0.23	0.60
AIC	109.81	108.91	97.94	93.81	109.80	110.09	110.55	108.16	109.27	100.48	108.46	93.28
*I_sar*	−0.01	−0.01	0.05	0.10	0.00	−0.01	−0.01	−0.05	0.00	−0.01	0.02	−0.06
MFD												
OLS												
Coef_ols	−0.68	0.11	−0.01	−0.02	−0.03	−0.07	0.00	−0.01	0.02	0.01	0.02	−0.01
r2_ols	0.09	0.03	0.07	0.08	0.02	0.14*	0.00	0.00	0.10	0.02	0.07	0.01
*I_ols*	−0.07	−0.17	−0.13	−0.09	−0.16	0.00	−0.06	−0.07	−0.18	−0.09	−0.17	−0.07
SAR												
Coef_sar	0.17	−0.34	−0.27	−0.29	−0.17	−0.37	0.02	−0.06	0.37	0.15	0.31	−0.12
AIC	110.89	107.77	109.11	108.76	110.92	106.40	111.92	111.80	107.05	111.12	108.65	111.40
*I_sar*	0.00	0.01	−0.07	−0.03	−0.06	0.04	0.00	0.00	0.01	0.01	0.01	−0.02
SESmfd												
OLS												
Coef_ols	−12.86	1.92	−0.08	−0.09	−0.29	−1.06	0.05	−0.20	0.23	0.40	0.43	0.04
r2_ols	0.14*	0.03	0.03	0.01	0.01	0.16*	0.00	0.01	0.06	0.05	0.08	0.00
*I_ols*	−0.07	−0.26	−0.08	−0.06	−0.13	0.05	−0.06	−0.10	−0.15	−0.15	−0.20	−0.05
SAR												
Coef_sar	0.19	−0.45	−0.12	−0.11	−0.05	−0.39	0.03	−0.11	0.28	0.27	0.36	0.04
AIC	110.62	104.89	111.36	111.50	111.43	105.74	111.88	111.53	109.12	109.40	107.60	111.85
*I_sar*	0.01	−0.02	−0.03	−0.01	−0.04	0.07	−0.01	−0.02	0.00	0.00	0.00	0.00

*Note*: Latitude and longitude are the value of the central location of the site. Coefficients (Coef), *r* ^2^, or AIC values were given. *p < .05; **p < .01.

Abbreviations: Altitude.HD, the vertical altitude difference (he unit is meter/m); Altitude.max, the highest altitude (the unit is meter/m); Altitude.mean, the mean altitude (the unit is meter/m); Altitude.min, the lowest altitude (the unit is meter/m); Area, the area of the site (the unit is km^2^); ASH, annual sunshine time (the unit is hour/h); AT, accumulated temperature (the unit is degrees Celsius/°C); MAP, mean annual precipitation (the unit is millimeters/mm); MAT, mean annual temperature (the unit is degrees Celsius/°C); Plant.ric, vascular plant species richness.

**FIGURE 2:**
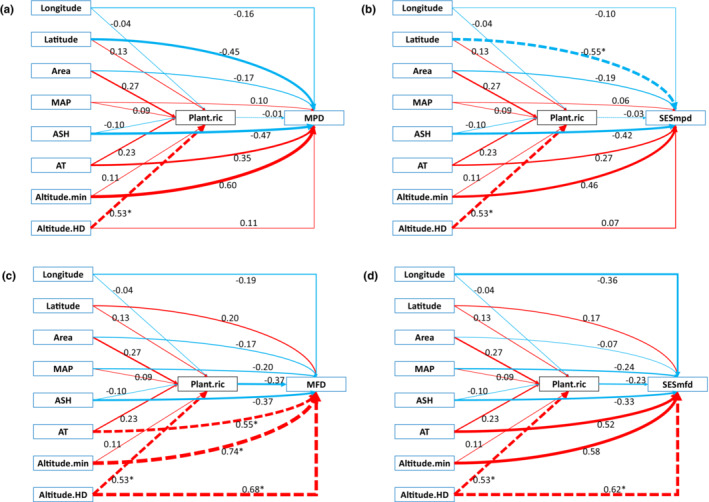
Results of structural equation model (SEM) examining the direct and indirect effects of longitude, latitude, area, mean annual precipitation (MAP), annual sunshine hours (ASH), accumulated temperature (AT), the lowest altitude (Altitude.min), vertical altitude difference (Altitude.HD), and plant species richness (Plant.ric) on phylogenetic structure (MPD, a, SESmpd, b), and functional structure (MFD, c, SESmfd, d). Red represents a positive relationship and green represents a negative relationship. Dashed lines indicate significant correlations. Standardized regression coefficients were given, and the strength of the effects was proportional to line width. *p < .05, **p < .01.

Phylogenetic structure of bird community was dispersed in the west (SESmpd > 0) which means interspecific competition plays a major role in community assemblage, and clustered in the east (SESmpd < 0) which means habitat filtration plays a major role (Table 1, Figure 2, Appendix S9). The functional structure of bird community was dispersed (SESmfd > 0) in all sites, and the degree of dispersion increases gradually from southeast to west and north (Table 1, Figure 2, Appendix S10). On the whole, interspecific competition is the main assembly processes of passerine resident bird community in the west and south, while that environmental filtration in the east and north of east Yunnan‐Kweichow Plateau.

SAR model was used to eliminate the spatial autocorrelation existed in the residuals of OLS regression between Plant.ric and SR, PD and FD (Table 1: Moran's *I* is 0.32*, 0.32*, 0.37*, respectively). By the OLS and SAR analysis, three diversity indices show similar pattern about the associations between bird species richness, phylogenetic structure, functional structure and each explanatory variable. Vascular plant richness (Plant.ric), habitat area (Area) and vertical elevation difference (Altitude.HD) are the three key factors to explain the diversity (SR, PD, FD), all of which have a significant positive correlation (Table 1). Area, Altitude.HD, latitude, and longitude are the key factors that have great influence on community phylogenetic structure index (MPD, SESmpd), while the factors that have great influence on community functional structure index (MFD, SESmfd) are mean annual precipitation (MAP), longitude and latitude. The results showed that the phylogenetic and functional structure of bird community changed in both longitude and latitude direction.

SEM analysis showed that longitude and Plant.ric had significant effects on SR, FD and PD, Area had a significant effect on SR, and the effect of Altitude.HD on bird diversity (SR, PD, FD) was indirectly caused by Plant.ric (Figure 1). Latitude had a significant effect on SESmpd, which is consistent with the results of OLS. AT, Altitude.min, and Altitude.HD had significant effects on MFD, Altitude.HD also had a significant effect on the SESmfd.

## 4 DISCUSSIONS

**FIGURE 3:**
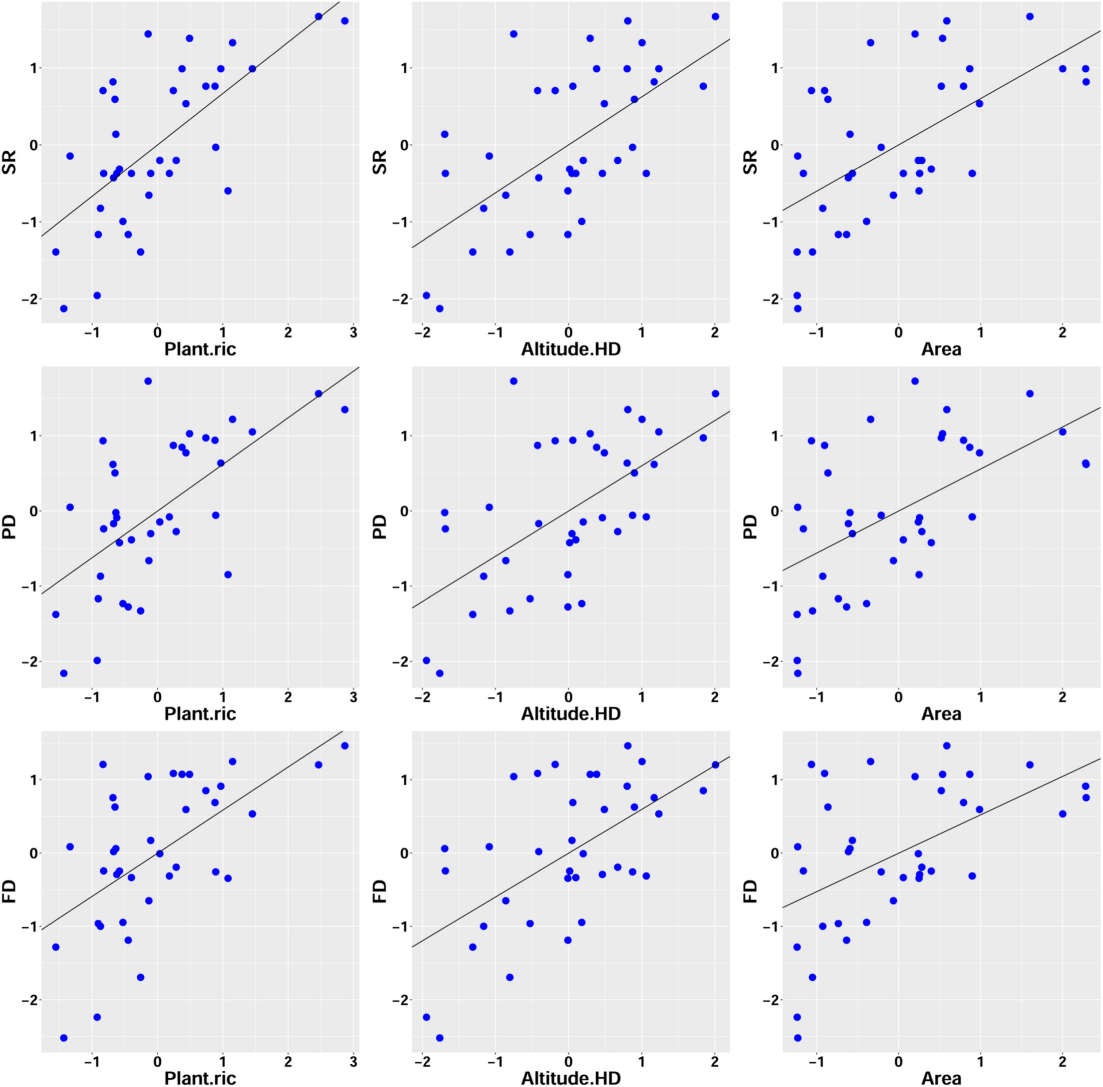
Scatter plots of resident passerine bird species richness (SR), phylogenetic diversity (PD) and functional diversity (FD) against their three most associated variables based on the SAR analysis.

In this paper, the effects of several habitat factors on bird diversity in different dimensions (SR, FD, and PD) and community structure were analyzed. The results showed that sites with rich vascular plant species, large area, and large vertical altitude difference had richer bird diversity (Table 1, Figure 3, Appendix S12).

### 4.1 Distribution pattern of bird diversity (SR, FD, and PD)

FJS has the highest SR value, ML has the highest PD value, DSH has the highest FD value, and Shilihetan park (SLHT) has the lowest value in all three indices (Appendix S8). Pearson correlation analysis showed that there was a significant positive correlation between PD, FD and SR respectively (Appendix S13), and these three indices have similar distribution patterns, which are relatively higher in the north, south and west, and lower in the middle and east. Generally speaking, National and provincial nature reserves have high bird diversity, such as Suoluo (SL), Xishui (XS), Dashahe (DSH) National Nature Reserves, they have high species richness (SR), and these species have distant genetic relationship and differentiated functional traits. In fact, FD and species richness (SR) are interrelated. The greater the number of species, the greater the change of the functional characteristics of the community, resulting in greater FD, however, there are still some differences between them, and the difference reflects the community functional structure and construction process, and so is the PD (Zhang & Fan, 2011).

### 4.2 The main ecological process of bird community construction

The combination of functional traits and phylogenetic information provides a potentially powerful strategy to reveal the construction mechanism of passerine resident bird community and the pattern of species coexistence in the east Yunnan‐Kweichow Plateau. Theoretically, these mechanisms can be summarized into two main set rules: habitat filtering and limiting similarity (Holdaway & Sparrow, 2006). Habitat filtering aggregates the characteristics of local communities by selecting species that are more similar than expected to respond to specific environmental conditions. In contrast, limiting similarity isolates the characteristics of local communities through interspecific competition, that is, excluding physiologically similar species, resulting in communities that are not as similar as accidentally expected (Calba et al., 2014; Holdaway & Sparrow, 2006). In this study, the main ecological process of bird Community construction revealed by functional and phylogenetic structure is not consistent, but the distribution pattern and changing trend of them from northeast to southwest are consistent. Therefore, it can be inferred that, the construction of passerine resident bird communities in the west and south of the East Yunnan‐Kweichow Plateau is mainly driven by interspecific competitive, while the process in the east and north is mainly driven by environmental filtration. This may be related to the topography of the East Yunnan‐Kweichow Plateau (high in the west and low in the east). Studies have shown that, when the environmental pressure is relatively large or the conditions are more extreme, closely related species that share similar traits or resource requirements coexist, resulting in phylogenetic clustering (Jarzyna et al., 2020; Machac et al., 2011; Si et al., 2017). As we know, the topography of the study area is high in the west and low in the east, the elevation difference increases gradually from east to west, and the habitat heterogeneity caused by the elevation difference will provide more food sources and living space for birds, therefore, the bird community in the west is dispersed driven by interspecific competition. In contrast, the habitat heterogeneity caused by altitude drop in east is low, although there are a few of nature reserves with good vegetation in the east, the type of vegetation is still relatively simple, so the bird community in the east is more concentrated under the action of habitat filtration. This reminds us of the importance of improving habitat heterogeneity when the topographical conditions of the east cannot be changed.

### 4.3 The main driving factors of community construction

This study showed that the three key factors affecting the distribution pattern of community diversity (SR, FD, and PD) were plant species richness (Plant.ric), area (Area) and altitude difference (Altitude.HD), which were positively correlated with each diversity index, respectively.

The relationship between Plant.ric and SR was verified by OLS, SAR and SEM analysis (Table 1, Table S9), which was consistent with previous research in China (Wang et al., 2020). Plant species richness is an important factor to measure the availability of bird food resources, the increase of plant species can directly promote the improvement of bird diversity and provide more niche space for birds, meanwhile, rich plants indirectly improve the diversity of small vertebrates and invertebrates, providing a rich food source for insectivorous or omnivorous bird (Ndang Ang et al., 2013).

Consistent with previous studies (Hanz et al., 2019; He et al., 2018; Wang et al., 2020; Zhang et al., 2020), this study also confirmed the significant effect of altitude on bird diversity (SR, FD, and PD). In natural ecosystems, large altitude drop is a representative feature of environmental heterogeneity, and the relationship between environmental heterogeneity and bird species richness has long been confirmed (Liang et al., 2018; Loss et al., 2009; Pellissier et al., 2012; Walter & Carsten, 2002). The distribution pattern of biota along the altitude gradient may be affected by number of physical and ecological factors, which may vary according to altitude, climate, habitat structure and resource availability (Lomolino, 2010). With the increase of altitude difference, environmental heterogeneity increased, and the habitat can provide more diversified habitat space and available food for birds, which was also verified in SEM analysis. In addition, SEM analysis also showed that the effect of Altitude.HD on bird diversity was mainly indirectly caused by vascular plant species richness (Plant.ric), which may be related to the change of vegetation conditions with altitude gradient.

Habitat area is an important factor affecting bird species diversity (Callaghan et al., 2019; Chang & Lee, 2016; Ding et al., 2016). Especially in urban green space, habitat area has been proved to be the most important factor affecting urban bird species diversity, and has a positive correlation with bird species diversity (Liu, 2019). Consistent with earlier studies, there was a positive correlation between habitat area and bird diversity index (SR, PD, and FD; Table 1, Appendix S12). The larger the habitat area was, the higher the habitat heterogeneity was, the more sufficient food can be provided for birds, and the more diversified habitat space was.

**FIGURE 4:**
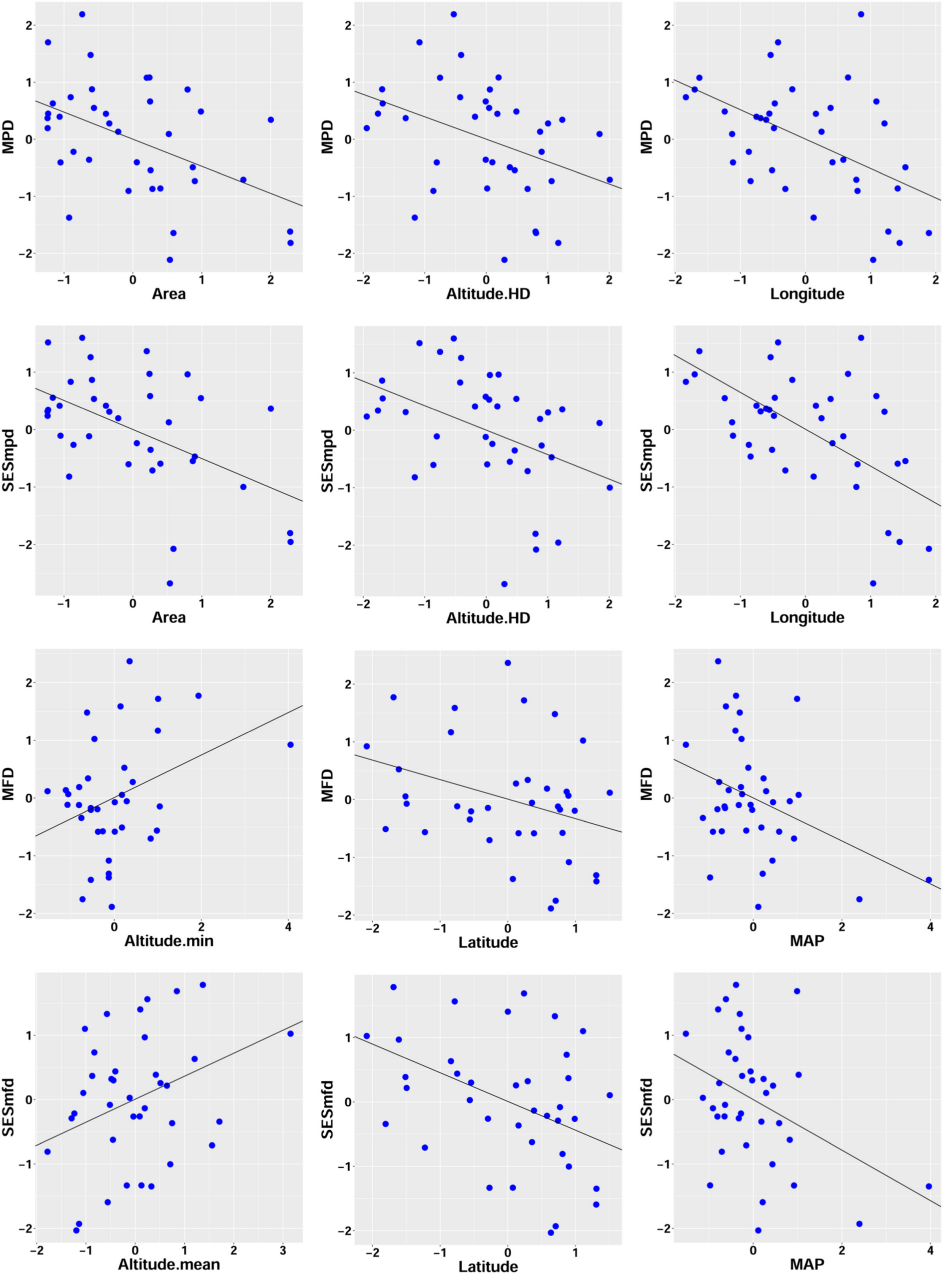
Scatter plots of community phylogenetic structure indices (MPD, SESmpd) and functional structure indices (MFD, SESmfd) against their three most associated variables based on the SAR analysis.

The phylogenetic structure of bird community is mainly affected by Area, Altitude.HD and Latitude. The OLS and SAR analysis showed that MPD and SESmpd was negatively correlated with Area, separately., that is, the larger the habitat area was, the more the phylogenetic structure tended to be clustered. Nature reserves with higher levels of protection usually have a larger area, with more complete vegetation and less human activities, while there are more forest specific birds, and habitat filtering may lead to community clustering, on the contrary, reserves with high human disturbance (such as Caohai and Bailidujuan nature reserve), or parks with low protection levels usually have relatively smaller areas and low habitat integrity, but high habitat heterogeneity, which can provide more diverse habitat and food sources for birds, and competitive exclusion may drive community divergence. Some related studies have shown that the FD and PD of birds in the secondary forest were significantly higher than those in the primary forest. The habitat filtering effect in the primary forest was stronger. There was a significant negative correlation between Altitude.HD and phylogenetic structure index (MPD, SESmpd; Table 1, Figure 4), that is, with the increase of altitude difference, bird community tended to be clustered, environmental filtration may be the main driving force of community construction, and bird community were mainly composed of young lineages, which was consistent with the early research (Dehling et al., 2014; Graham et al., 2009; Machac et al., 2011). This diversity pattern was usually attributed to the obvious differences in vegetation types along the altitude gradient.

The functional structure of community is mainly affected by MAP and Altitude.HD. MAP was negatively correlated with MFD, and SESmfd. With the increase of rainfall, the functional structure of birds became more concentrated, the functional traits of species in the community tended to be similar, and habitat filtration plays a more important role. Climate is one of the main determinants of species distribution limitations (Che et al., 2018). The complex effects of climatic factors on bird diversity and distribution pattern have been confirmed (Che et al., 2018; Hawkins et al., 2005; Li et al., 2013; Liang et al., 2020; Santillan et al., 2019; Wang et al., 2020; Zhang et al., 2018). This is in accordance with the finding of an Australian study (Remeš & Harmáčková, 2018) that the wetter the climate in Australia, the higher the bird species richness, and in the wetter and more productive northern and eastern Australia, the bird phylogenetic structure was more clustered. In addition, domestic studies have shown that the phylogenetic and functional structure of birds were also most clustered in the southwest region with moderate precipitation (Wang et al., 2020). Precipitation is an important factor affecting the gradient of biodiversity in most parts on earth (Hawkins et al., 2003), its effect on bird diversity may be indirectly caused by plant species richness (Brown, 1981), but it has not been effectively verified in SEM analysis. In addition, in the forest areas with higher altitude, the harsh climatic conditions may also cause direct metabolic effects and indirect food resource restrictions on some birds, resulting in that only birds adapted to cold and humid conditions can survive (He et al., 2018). Some studies have shown that high‐altitude ecosystems are particularly vulnerable to ecological disturbance and lead to loss of function in the future (Jarzyna et al., 2020).

## 5 CONCLUSION

This paper attempted to analyze the distribution pattern of passerine resident bird diversity in the East Yunnan‐Kweichow Plateau and to explore the main ecological processes and driving factors of bird community construction. The results show that SR, FD, and PD have similar distribution patterns, and are mainly affected by plant species richness, altitude difference and habitat area. As a result of the joint action of multiple habitat factors, the community construction in the west and south of this area is mainly driven by interspecific competitive, while environmental filtration in the east and north. However, the types of habitat factors used in this study are limited, so it is necessary to collect more influencing factors for in‐depth analysis.

## AUTHOR CONTRIBUTIONS

**Haibo Zhang:** Data curation (equal); formal analysis (equal); investigation (equal); methodology (equal); software (equal); writing – original draft (lead). **Lingbin Yan:** Methodology (equal); software (equal); writing – review and editing (equal). **Lifei Yu:** Project administration (lead); writing – review and editing (lead). **Haijun Su:** Conceptualization (equal); methodology (equal); writing – review and editing (equal). **Canshi Hu:** Conceptualization (equal); software (equal); writing – review and editing (equal). **Mingming Zhang:** Conceptualization (equal); writing – review and editing (equal). **Zhihong Kong:** Conceptualization (equal); writing – review and editing (equal).

## Supporting information


Appendix S1.

Appendix S2.

Appendix S3.

Appendix S4.

Appendix S5.

Appendix S6.

Appendix S7.

Appendix S8.

Appendix S9.

Appendix S10.

Appendix S11.

Appendix S12.

Appendix S13.

## Data Availability

The data that supports the findings of this study are available in the [Link], [Link], [Link], [Link], [Link], [Link], [Link], [Link], [Link], [Link], [Link], [Link], [Link] of this article.
