# Supplementary material for: The diversity of resident passerine bird in the East Yunnan‐Kweichow Plateau is closely related to plant species richness, vertical altitude difference and habitat area
Source: Ecol Evol. 2023 Jan 17;13(1):e9735. doi: 10.1002/ece3.9735 (PMC9843479; doi:10.1002/ece3.9735)
Supplement: Supplementary file 1 — Appendix S1. [file ECE3-13-e9735-s009.docx]

**Appendix S1** **All 37study sites and data sources in East Yunnan-Kweichow Plateau**

| **No** | **Sites** | **Abbreviation** | **Administrative region** | **Type** | **Management level** | **Bird data sources** | **Environmental factors data sources** |
| --- | --- | --- | --- | --- | --- | --- | --- |
| 1 | Aha Lake National Wetland Park | AHH | Guiyang City | Park | national | Zhang, H.Sun, X.Li, G.Wu, Z.Kuang, Z.Su, H.Xia, F., &Tan, H., 2020. Diversity analysis of bird communities in aha lake national wetland Park,Guiyang,China. Chinese Journal of Wildlife, 41, 626-640. 10.19711/j.cnki.issn2310-1490.2020.03.010. | Zhang, H.Sun, X.Li, G.Wu, Z.Kuang, Z.Su, H.Xia, F., &Tan, H., 2020. Diversity analysis of bird communities in aha lake national wetland Park,Guiyang,China. Chinese Journal of Wildlife, 41, 626-640. 10.19711/j.cnki.issn2310-1490.2020.03.010. |
|  |  |  |  |  |  |  | Wang, L.Li, J.Kong, Z.Su, Y.Liu, Y.Chen, X.Xie, Y.Wang, Y., &Jin, H., 2019. Dynamic evolution of landscape pattern in Aha Lake National Wetland Park in recent 20 Years. Journal of Anhui Normal University(Natural Science）, 42, 40-45. 10.14182/J.cnki.1001-2443.2019.01.007. |
| 2 | Badashan Nature reserve | BDS | Panzhou City | Nature reserve | county level | Zhu, J. (2013). Scientific investigation and research on the Badashan Nature Reserve in Panxian County, Guizhou Province. Beijing: China Forestry Publishing House. | Zhu, J. (2013). Scientific investigation and research on the Badashan Nature Reserve in Panxian County, Guizhou Province. Beijing: China Forestry Publishing House. |
| 3 | Basha Nature reserve | BS | Congjiang County | Nature reserve | county level | Luo, Y. (2018). Scientific investigation and research on Basha Tuomiao Dashan Nature Reserve in Congjiang County, Guizhou Province. | Luo, Y. (2018). Scientific investigation and research on Basha Tuomiao Dashan Nature Reserve in Congjiang County, Guizhou Province. |
|  |  |  |  |  |  |  | Lu, X.Yang, C.Yang, L.Yang, B., &Li, M., 2018. Study on the Flora of Lycopsida and Pteridophyte in Basha Nature Reserve, Congjiang County. Guizhou Forestry Science and Technology, 46, 5. |
| 4 | Bailidujuan Nature reserve | BLDJ | Qianxi County, Dafang County | Nature reserve | provincial | Huang, X. (2016). Study on the relationship between the vertebrate resources and environmental change in Guizhou Baili Rodrodendron Nature Reserve. In. Vol. Master. Guiyang: Guizhou University. | Huang, X. (2016). Study on the relationship between the vertebrate resources and environmental change in Guizhou Baili Rodrodendron Nature Reserve. In. Vol. Master. Guiyang: Guizhou University. |
|  |  |  |  |  |  |  | Li, W.Zhang, H.Wu, D.Zhang, J., &Jia, Z., 2020. Study on the diversity and stability of plant communities in Baili hododendron scenic area in Guizhou Province. Forest Resources Management, 0, 6. 10.13466/j.cnki.lyzygl.2020.02.018. |
| 5 | Baimianshui Nature reserve | BMS | Meitan County | Nature reserve | provincial | Zhang, H. (2006). Comprehensive scientific investigation of Meitan Baimianshui Nature Reserve. Guiyang: Guizhou Science and Technology Press. | Zhang, H. (2006). Comprehensive scientific investigation of Meitan Baimianshui Nature Reserve. Guiyang: Guizhou Science and Technology Press. |
| 6 | Baiqing Nature reserve | BQ | Tongzi County | Nature reserve | municipal | Yan, Y.Kuang, Z.Jiang, Y.Chen, D.Chen, S., &Zhang, G., 2017. Summer bird resources of Tongzi Huanglianbaijing Nature Reserve in Guizhou Province. Chinese Journal of Wildlife, 38, 265-272. 10.3969/j.issn.1000-0127.2017.02.018. | Yu, L., 2002. Study on diversity of evergreen and deciduous broadleaf forest in Baiqing karst platform area in Guizhou province. Guizhou Science, 20, 5. CNKI:SUN:GZKX.0.2002-02-004. |
|  |  |  |  |  |  | Lan, K. (1994). Scientific investigation of Baiqing karst dainohara forest area. Guiyang: Guizhou Nationalities Publishing House. | Lan, K. (1994). Scientific investigation of Baiqing karst dainohara forest area. Guiyang: Guizhou Nationalities Publishing House. |
|  |  |  |  |  |  |  | Yan, Y.Kuang, Z.Jiang, Y.Chen, D.Chen, S., &Zhang, G., 2017. Summer bird resources of Tongzi Huanglianbaijing Nature Reserve in Guizhou Province. Chinese Journal of Wildlife, 38, 265-272. 10.3969/j.issn.1000-0127.2017.02.018. |
| 7 | Caohai Nature reserve | CH | Weining County | Nature reserve | national | Zhang, H.Li, M., &Yao, S. (2007). Caohai Research. Guiyang: Guizhou Science and Technology Press. | Zhang, H.Li, M., &Yao, S. (2007). Caohai Research. Guiyang: Guizhou Science and Technology Press. |
|  |  |  |  |  |  | Caohai National Nature Reserve Management Committee, G. P. (2017). Investigation and Research Report on vertebrate Diversity in Caohai National Nature Reserve. | Caohai National Nature Reserve Management Committee, G. P. (2017). Investigation and Research Report on vertebrate Diversity in Caohai National Nature Reserve. |
| 8 | Dashanhe Nature reserve | DSH | Daozhen County | Nature reserve | national | Li, Q.Gou, W.Ji, C.Xiao, W.Chen, S., &Xiao, Z., 2022. Camera-trapping survey of mammals and birds in the Guizhou Dashahe National Nature Reserve. Acta Theriologica Sinica, 42, 10. 10.16829/j.slxb.150536. | Li, Q.Gou, W.Ji, C.Xiao, W.Chen, S., &Xiao, Z., 2022. Camera-trapping survey of mammals and birds in the Guizhou Dashahe National Nature Reserve. Acta Theriologica Sinica, 42, 10. 10.16829/j.slxb.150536. |
|  |  |  |  |  |  | Xie, S.Yu, L., &Zhou, Q. (2006). Background resources of Dashahe nature reserve. Guiyang: Guizhou Science and Technology Press. | Xie, S.Yu, L., &Zhou, Q. (2006). Background resources of Dashahe nature reserve. Guiyang: Guizhou Science and Technology Press. |
| 9 | Fanjingshan Nature reserve | FJS | Jiangkou County, Yinjiang County, Songtao County | Nature reserve | national | Kuang, Z., &Niu, K. (2016). Birds of Fanjing Mountain. Guiyang: Guizhou Science and Technology Press. | Kuang, Z., &Niu, K. (2016). Birds of Fanjing Mountain. Guiyang: Guizhou Science and Technology Press. |
|  |  |  |  |  |  |  | Zhang, M.Yang, C.Wang, C.Wang, J.Hu, C.Lei, X.Shi, L.Su, H., &Li, J., 2019. Camera-trapping survey on mammals and birds in Fanjingshan National Nature Reserve, Guizhou, China. Biodiversity Science, 813-818. 10.17520/biods.2019131. |
| 10 | Fodingshan Nature reserve | FDS | Shiqian County | Nature reserve | national | Yu, L.Su, H.An, M.Yang, R., &Zhang, H. (2016). Biodiversity of Foding Mountain in Guizhou: research on the Wuling Mountain-Miaoling Mountain node of biodiversity conservation priority areas in China. Guiyang: Guizhou Science and Technology Press. | Yu, L.Su, H.An, M.Yang, R., &Zhang, H. (2016). Biodiversity of Foding Mountain in Guizhou: research on the Wuling Mountain-Miaoling Mountain node of biodiversity conservation priority areas in China. Guiyang: Guizhou Science and Technology Press. |
| 11 | Fuyan Nature reserve | FY | Zheng‘an County | Nature reserve | municipal | Zhang, H. (2003). A scientific investigations in Fuyan nature reserve. Guiyang: Guizhou Science and Technology Press. | Zhang, H. (2003). A scientific investigations in Fuyan nature reserve. Guiyang: Guizhou Science and Technology Press. |
| 12 | Gongtong Nature reserve | GT | Nayong County | Nature reserve | provincial | Deng, L.Jingcheng, R., &Shang, J. (2013). Scientific investigation and research on Nayong Dove Tree Nature Reserve in Guizhou Province. Beijing: China Forestry Publishing House. | Deng, L.Jingcheng, R., &Shang, J. (2013). Scientific investigation and research on Nayong Dove Tree Nature Reserve in Guizhou Province. Beijing: China Forestry Publishing House. |
| 13 | Houshuihe Nature reserve | HSH | Suiyang County | Nature reserve | county level | Zhu, K.Xiang, G.Dang, A.Xu, W., &Feng, P., 2022. Survey and assessment on birds in Houshuihe Lake of Houshuihe Nature Reserve,Suiyang Counnty,Guizhou Province. Environmental Protection and Technology, 22, 32-35. 10.3969/j.issn.1674-0254.2016.01.007. | Zhu, K.Xiang, G.Dang, A.Xu, W., &Feng, P., 2022. Survey and assessment on birds in Houshuihe Lake of Houshuihe Nature Reserve,Suiyang Counnty,Guizhou Province. Environmental Protection and Technology, 22, 32-35. 10.3969/j.issn.1674-0254.2016.01.007. |
| 14 | Kaili University | KLXY | Kaili City | Campus | none | Luo, Z.Niu, J.Li, X.Zhang, W., &Meng, L., 2012. Avian biodiversity in campus of Kaili University, Guizhou Province. Journal of Hubei University(Natural Science), 034, 292-297. 10.3969/j.issn.1000-2375.2012.03.011. | Luo, Z.Niu, J.Li, X.Zhang, W., &Meng, L., 2012. Avian biodiversity in campus of Kaili University, Guizhou Province. Journal of Hubei University(Natural Science), 034, 292-297. 10.3969/j.issn.1000-2375.2012.03.011. |
| 15 | Kuankuoshui Nature reserve | KKS | Suiyang County | Nature reserve | national | Yu, L.Chen, G., &Yu, D. (2018). Research on biodiversity conservation in Kuankuoshui National Nature Reserve in Guizhou. Beijing: China Forestry Publishing House. | Yu, L.Chen, G., &Yu, D. (2018). Research on biodiversity conservation in Kuankuoshui National Nature Reserve in Guizhou. Beijing: China Forestry Publishing House. |
| 16 | Laoshechong Nature reserve | LSC | Majiang County | Nature reserve | municipal | Zhang, H. (2003). Scientific investigation of Laoshechong Nature Reserve. Guiyang: Guizhou Science and Technology Press. | Zhang, H. (2003). Scientific investigation of Laoshechong Nature Reserve. Guiyang: Guizhou Science and Technology Press. |
| 17 | Leigongshan Nature reserve | LGS | Leishan County, Taijiang County, Jianhe County, Rongjiang County | Nature reserve | national | Zhang, H., &Zhang, X. (2007). Research on biodiversity of Leigongshan National Nature Reserve. Guiyang: Guizhou Science and Technology Press. | Zhang, H., &Zhang, X. (2007). Research on biodiversity of Leigongshan National Nature Reserve. Guiyang: Guizhou Science and Technology Press. |
|  |  |  |  |  |  | Wang, Z.Li, P.Li, Y., &Xie, Z., 2015. New bird records for Leigongshan National Nature Reserve, Guizhou, China. Chinese Journal of Wildlife, 36, 118-120. 10.19711/j.cnki.issn2310-1490.2015.01.024. |  |
|  |  |  |  |  |  | Xie, Z.Li, Y.Li, X., &Yang, S., 2014. New record of birds in Leigongshan Nature Reserve. Agricultural Technology Service, 31, 2. 10.3969/j.issn.1004-8421.2014.07.128. |  |
|  |  |  |  |  |  | Huang, S.Li, D., &Wang, Z., 2020. New record of birds in Leigongshan Nature Reserve - Brown-eared Eagle and Crested Eagle. Agricultural Technology Service, 37, 2. |  |
|  |  |  |  |  |  | Hou, D.Gu, X.Zhang, Q., &Yao, L., 2020. Infrared camera monitoring of wild animals in Leigongshan Nature Reserve. South China Agriculture, 14, 3. 10.19415/j.cnki.1673-890x.2020.24.091. |  |
| 18 | Lenshuihe Nature reserve | LSH | Jinsha County | Nature reserve | county level | Peng, L., &Liao, D., 2008. Species diversity and conservation in Lengshui River Nature Reserve in Guizhou Province. Central South Forest Inventory and Planning, 27, 4. 10.3969/j.issn.1003-6075.2008.02.017. | Peng, L., &Liao, D., 2008. Species diversity and conservation in Lengshui River Nature Reserve in Guizhou Province. Central South Forest Inventory and Planning, 27, 4. 10.3969/j.issn.1003-6075.2008.02.017. |
|  |  |  |  |  |  | Luo, Y. (2018). Comprehensive scientific investigation report on Jinsha Lengshuihe Nature Reserve in Guizhou Province. | Luo, Y. (2018). Comprehensive scientific investigation report on Jinsha Lengshuihe Nature Reserve in Guizhou Province. |
| 19 | Longyin Nature reserve | LY | Pu'an County | Nature reserve | county level | Yang, W., &Ran, J. (2019). Comprehensive scientific investigation of Longyin broad-leaved forest state-level nature reserve in Pu'an, Guizhou. Beijing: China Forestry Publishing House. | Yang, W., &Ran, J. (2019). Comprehensive scientific investigation of Longyin broad-leaved forest state-level nature reserve in Pu'an, Guizhou. Beijing: China Forestry Publishing House. |
| 20 | Mayanghe Nature reserve | MYH | Yanhe County, Wuchuan County | Nature reserve | national | Gou, G.Wei, N., &Xie, S. (2017). Research on biodiversity of Mayanghe National Nature Reserve in Guizhou. Guiyang: Guizhou Science and Technology Press. | Gou, G.Wei, N., &Xie, S. (2017). Research on biodiversity of Mayanghe National Nature Reserve in Guizhou. Guiyang: Guizhou Science and Technology Press. |
|  |  |  |  |  |  | Yang, X.Wu, A.Zou, Q.Li, G.Zhang, M.Hu, C., &Su, H., 2020. Field monitoring of mammals and birds using infrared cameras in Mayanghe National Nature Reserve, Guizhou, China. Biodiversity Science, 28, 7. 10.17520/biods.2019306. | Yang, X.Wu, A.Zou, Q.Li, G.Zhang, M.Hu, C., &Su, H., 2020. Field monitoring of mammals and birds using infrared cameras in Mayanghe National Nature Reserve, Guizhou, China. Biodiversity Science, 28, 7. 10.17520/biods.2019306. |
| 21 | Maolan Nature reserve | ML | Libo County | Nature reserve | national | Kuang, Z., &Yao, Z. (2020). Birds of Maolan, China. Beijing: Science Press. | Kuang, Z., &Yao, Z. (2020). Birds of Maolan, China. Beijing: Science Press. |
|  |  |  |  |  |  | Xiong, K. (2006). Cone karst ecological processes and biodiversity of Libo. Guiyang: Guizhou People's Press. | Xiong, K. (2006). Cone karst ecological processes and biodiversity of Libo. Guiyang: Guizhou People's Press. |
| 22 | Minghu National Wetland Park | MH | Liupanshui City | Park | national | Chen, H.Li, S.Xiong, R., &Tian, Y., 2013. An Iinvestigation of vertebrate resources in Minghu National Wetland Park of Liupanshui, Guizhou Province. Journal of Liupanshui Teachers College, 25, 1-20. 10.3969/j.issn.1671-055X.2013.06.001. | Chen, H.Li, S.Xiong, R., &Tian, Y., 2013. An Iinvestigation of vertebrate resources in Minghu National Wetland Park of Liupanshui, Guizhou Province. Journal of Liupanshui Teachers College, 25, 1-20. 10.3969/j.issn.1671-055X.2013.06.001. |
|  |  |  |  |  |  |  | Qin, Q.Dai, W., &Liu, X., 2013. Health assessment on artificial wetland of city ecosystem in Wumeng Mountain area ——case of Minghu National wetland Park in Liupanshui City. Journal of Hydroecology, 34, 43-46. |
| 23 | Nangong Nature reserve | NGO | Taijiang County | Nature reserve | municipal | Zhang, H. (2003). Scientific expedition to Nangong Nature Reserve. Guiyang: Guizhou Science and Technology Press. | Zhang, H. (2003). Scientific expedition to Nangong Nature Reserve. Guiyang: Guizhou Science and Technology Press. |
| 24 | Nan'gan Nature reserve | NGA | Dejiang County | Nature reserve | provincial | Kuang, Z., &Li, Z. (2016). Bird survey report of Nangan Nature Reserve of Guizhou Province. | Yao, Y.Xu, F.Liu, D., &Zhi, H., 2019. Species and faunal analysis on insects in Guizhou Dejiang Nangan Nature Reserve. Journal of Zhejiang Forestry Science and Technology, 39, 5. CNKI:SUN:ZJLK.0.2019-04-009. |
|  |  |  |  |  |  |  | Kuang, Z., &Li, Z. (2016). Bird survey report of Nangan Nature Reserve of Guizhou Province. |
| 25 | Pogang Nature reserve | PG | Xingyi City | Nature reserve | municipal | Zhang, H.Long, Q., &Liao, D. (2006). Compilation of comprehensive scientific investigation of Pogang Nature Reserve in Xingyi City. Guiyang: Guizhou Science and Technology Press. | Zhang, H.Long, Q., &Liao, D. (2006). Compilation of comprehensive scientific investigation of Pogang Nature Reserve in Xingyi City. Guiyang: Guizhou Science and Technology Press. |
| 26 | Qiannan Normal University for Nationalities | QNSY | Duyun City | Campus | none | Yang, C.Luo, Q.Zhao, W., &Wang, F., 2020. Investigation on birds in the campus of Qiannan Normal University for Nationalities and its surrounding greenbelts. Journal of Anhui Agricultural Sciences, 48, 98-101, 142. 10.3969/j.issn.0517-6611.2020.16.026. | Yang, C.Luo, Q.Zhao, W., &Wang, F., 2020. Investigation on birds in the campus of Qiannan Normal University for Nationalities and its surrounding greenbelts. Journal of Anhui Agricultural Sciences, 48, 98-101, 142. 10.3969/j.issn.0517-6611.2020.16.026. |
| 27 | Shilihetan Urban Wetland Park | SLHT | Guiyang City | Park | none | Wang, Y.Zhong, G.Song, B.Pan, C.Zhang, Y., &Wang, L., 2019. Preliminary study on Huaxi national urban wetland park bird diversity. Journal of Guizhou Normal University ( Natural Sciences), 37, 59-64. 10.16614/j.gznuj.zrb.2019.01.011. | Wang, Y.Zhong, G.Song, B.Pan, C.Zhang, Y., &Wang, L., 2019. Preliminary study on Huaxi national urban wetland park bird diversity. Journal of Guizhou Normal University ( Natural Sciences), 37, 59-64. 10.16614/j.gznuj.zrb.2019.01.011. |
| 28 | Siyetun Nature reserve | SYT | Sinan County | Nature reserve | provincial | Zhu, J.Chen, D., &Xie, J. (2017). Scientific survey of Siyetun investigation of Sinan Nature Reserve of Sinan county of Guizhou Province. Beijing: China Forestry Publishing House. | Zhu, J.Chen, D., &Xie, J. (2017). Scientific survey of Siyetun investigation of Sinan Nature Reserve of Sinan county of Guizhou Province. Beijing: China Forestry Publishing House. |
| 29 | Sutie Nature reserve | ST | Wangmo County | Nature reserve | county level | LUO, Y., &LIU, L. (2010). Scientific investigation of Wangmo Cycad Nature Reserve in Guizhou Province. Guiyang: Guizhou Science and Technology Press. | LUO, Y., &LIU, L. (2010). Scientific investigation of Wangmo Cycad Nature Reserve in Guizhou Province. Guiyang: Guizhou Science and Technology Press. |
| 30 | Suoluo Nature reserve | SL | Chishui City | Nature reserve | national | Deng, H. (2015). Biodiversity of Chishui Cypress National Nature Reserve in Guizhou. Beijing: Science Press. | Deng, H. (2015). Biodiversity of Chishui Cypress National Nature Reserve in Guizhou. Beijing: Science Press. |
|  |  |  |  |  |  | Yin, X. (2013). Research on vertebrate diversity in Chishui cypress national nature reserve. In. Vol. Master. Chongqing: Southwest University. |  |
|  |  |  |  |  |  | Wang, C.Zhou, D.Liang, S.Su, H.Hu, C., &Zhang, M., 2019. Camera-trapping survey on mammals and birds in Guizhou Chishui Alsophila National Nature Reserve. Biodiversity Science, 27, 6. 10.17520/biods.2019220. |  |
| 31 | Taipingshan Nature reserve | TPS | Liping County | Nature reserve | municipal | Zhang, H.Li, M., &Deng, J. (2006). Comprehensive scientific investigation of Liping Taiping Mountain Nature Reserve. Guiyang: Guizhou Science and Technology Press. | Zhang, H.Li, M., &Deng, J. (2006). Comprehensive scientific investigation of Liping Taiping Mountain Nature Reserve. Guiyang: Guizhou Science and Technology Press. |
| 32 | Wanfoshan Nature reserve | WFS | Fenggang County | Nature reserve | county level | Hu, C.Yang, X., &Tian, Q. (2018). Investigation report on bird resources in Wanfoshan nature reserve in Fenggang County. | Hu, C.Yang, X., &Tian, Q. (2018). Investigation report on bird resources in Wanfoshan nature reserve in Fenggang County. |
|  |  |  |  |  |  |  | Li, S.Yu, K.An, M.Huang, S., &Luo, C., 2020. Fish survey and evaluation in Nature Reserves of Fenggang Country of Guizhou. Guizhou Journal of Animal Husbandry & Veterinary Medicine, 44, 5. 10.3969/j.issn.1007-1474.2020.01.007. |
|  |  |  |  |  |  |  | Su, J.He, L.Wei, M., &Sun, Z., 2013. Analysis on flora of bryophytes on rock in Wanfoshan Provincial Forest Park of Guizhou. Hubei Agricultural Science, 52, 2788-2791. 10.3969/j.issn.0439-8114.2013.12.015. |
| 33 | Xishui Nature reserve | XS | Xishui County | Nature reserve | national | Luo, Y.Liu, L., &Yang, R. (2012). Scientific investigation and research national nature reserve of the subtropical evergreen broad-leaved forest in Xishui County, Guizhou Province. Guiyang: Guizhou Science and Technology Press. | Luo, Y.Liu, L., &Yang, R. (2012). Scientific investigation and research national nature reserve of the subtropical evergreen broad-leaved forest in Xishui County, Guizhou Province. Guiyang: Guizhou Science and Technology Press. |
|  |  |  |  |  |  | Mu, J.Wang, J.Zhang, L.Li, Y.Li, Z., &Su, H., 2019. Field monitoring using infrared cameras and activity rhythm analysis on mammals and birds in Xishui National Nature Reserve, Guizhou, China. Biodiversity Science, 27, 6. 10.17520/biods.2018347. |  |
| 34 | Yangxi Nature reserve | YX | Yinjiang County | Nature reserve | provincial | Zhu, J.Jiang, Y., &Zhang, A. (2017). Comprehensive scientific investigation and research on Yangxi Nature Reserve of Yinjiang County in Guizhou. Beijing: China Forestry Publishing House. | Zhu, J.Jiang, Y., &Zhang, A. (2017). Comprehensive scientific investigation and research on Yangxi Nature Reserve of Yinjiang County in Guizhou. Beijing: China Forestry Publishing House. |
| 35 | Yezhong Nature reserve | YZ | Shuicheng County | Nature reserve | municipal | Ke, X. (2019). Report on Vertebrate Diversity in Yezhong Nature Reserve of Liupanshui City in Guizhou Province. | Ke, X. (2019). Report on Vertebrate Diversity in Yezhong Nature Reserve of Liupanshui City in Guizhou Province. |
|  |  |  |  |  |  | Province, E. P. B. O., &City, E. P. B. O. (1990). Scientific investigation of Yezhong Black Langur Nature Reserve of Liupanshui City in Guizhou Province. Guiyang: Guizhou Nationalities Publishing House. | Province, E. P. B. O., &City, E. P. B. O. (1990). Scientific investigation of Yezhong Black Langur Nature Reserve of Liupanshui City in Guizhou Province. Guiyang: Guizhou Nationalities Publishing House. |
| 36 | Yueliangshan Nature reserve | YLS | Rongjiang County | Nature reserve | municipal | Luo, Y.Long, D.Li, M.Yang, Y.Shi, W., &Chen, Z. (2019). Scientific investigation and study of Guizhou Yueliangshan Nature Reserve in Guizhou Province. Beijing: China Forestry Publishing House. | Luo, Y.Long, D.Li, M.Yang, Y.Shi, W., &Chen, Z. (2019). Scientific investigation and study of Guizhou Yueliangshan Nature Reserve in Guizhou Province. Beijing: China Forestry Publishing House. |
| 37 | Yuntaishan Nature reserve | YTS | Shibin County | Park | none | Luo, Z.Liu, L.Wang, Y.Niu, J.Liu, L., &Li, L., 2013. Characteristics of the avian communities at Mt.Yuntaishan of Guizhou Province, China. Journal of East China Normal University(Natural Science), 43-52. 10.3969/j.issn.1000-5641.2013.05.006. | Wang, Z., &Zhang, C., 2010. A study on biomass of bryophyte communities at the karst forest ecosystem in Mt.Yuntai , Guizhou province. Journal of Guizhou Normal University(Natural Sciences), 28, 4. 10.3969/j.issn.1004-5570.2010.04.020. |
|  |  |  |  |  |  |  | Wang, C., &Chen, L., 1996. A study on the flora of seed plants of Yuntai Mountain in Guizhou Province. Journal fo Guizhou Normal University (Natural Science), 14, 6. |
|  |  |  |  |  |  |  | Luo, Z.Liu, L.Wang, Y.Niu, J.Liu, L., &Li, L., 2013. Characteristics of the avian communities at Mt.Yuntaishan of Guizhou Province, China. Journal of East China Normal University(Natural Science), 43-52. 10.3969/j.issn.1000-5641.2013.05.006. |
