# Supplementary figures and images for: The diversity of resident passerine bird in the East Yunnan‐Kweichow Plateau is closely related to plant species richness, vertical altitude difference and habitat area
Source: Ecol Evol. 2023 Jan 17;13(1):e9735. doi: 10.1002/ece3.9735 (PMC9843479; doi:10.1002/ece3.9735)

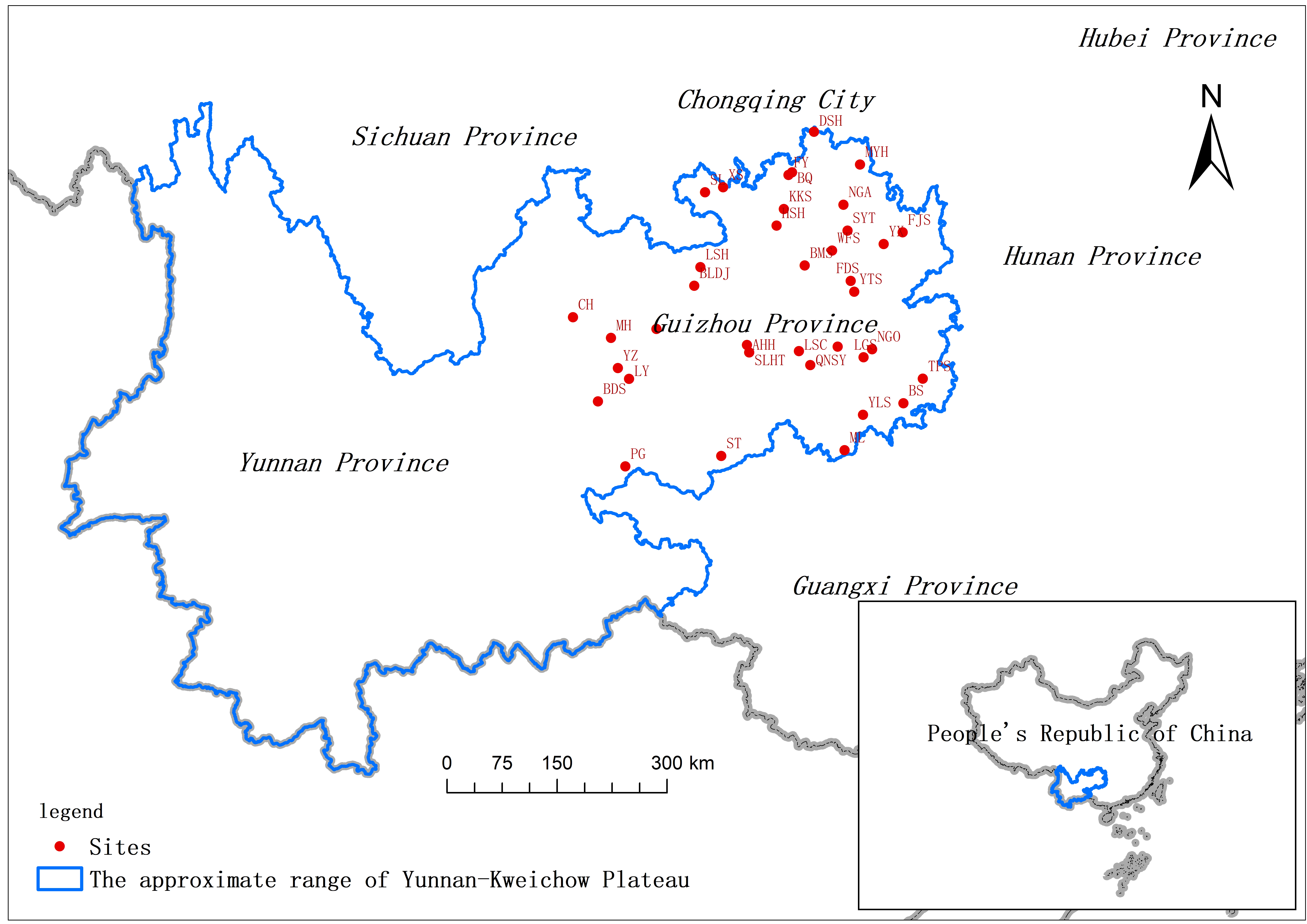

Supplement: Supplementary file 2 — Appendix S2. [file ECE3-13-e9735-s002.jpg]

length

66.91

0

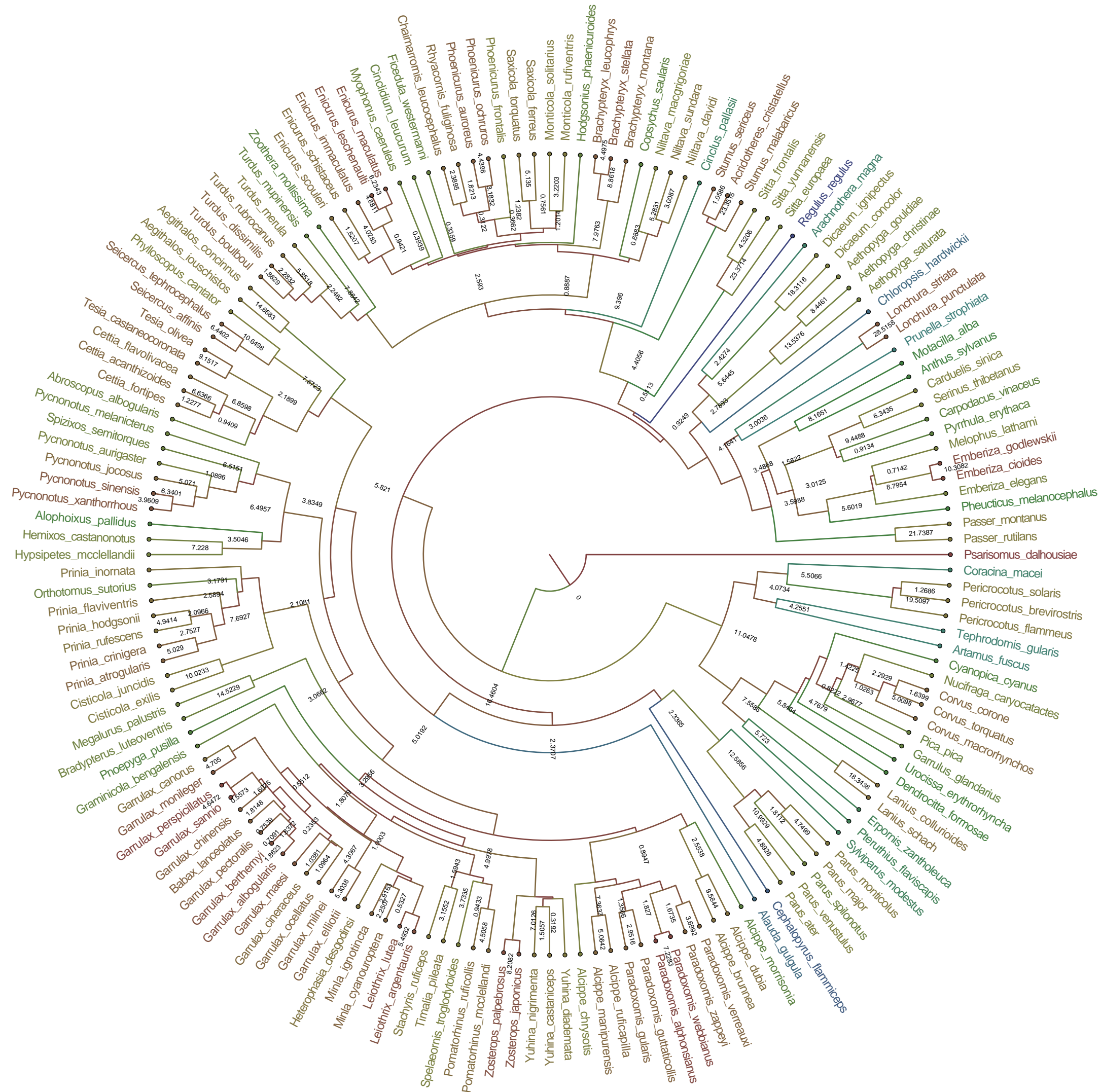

Supplement: Supplementary file 4 — Appendix S4. [file ECE3-13-e9735-s006.pdf]

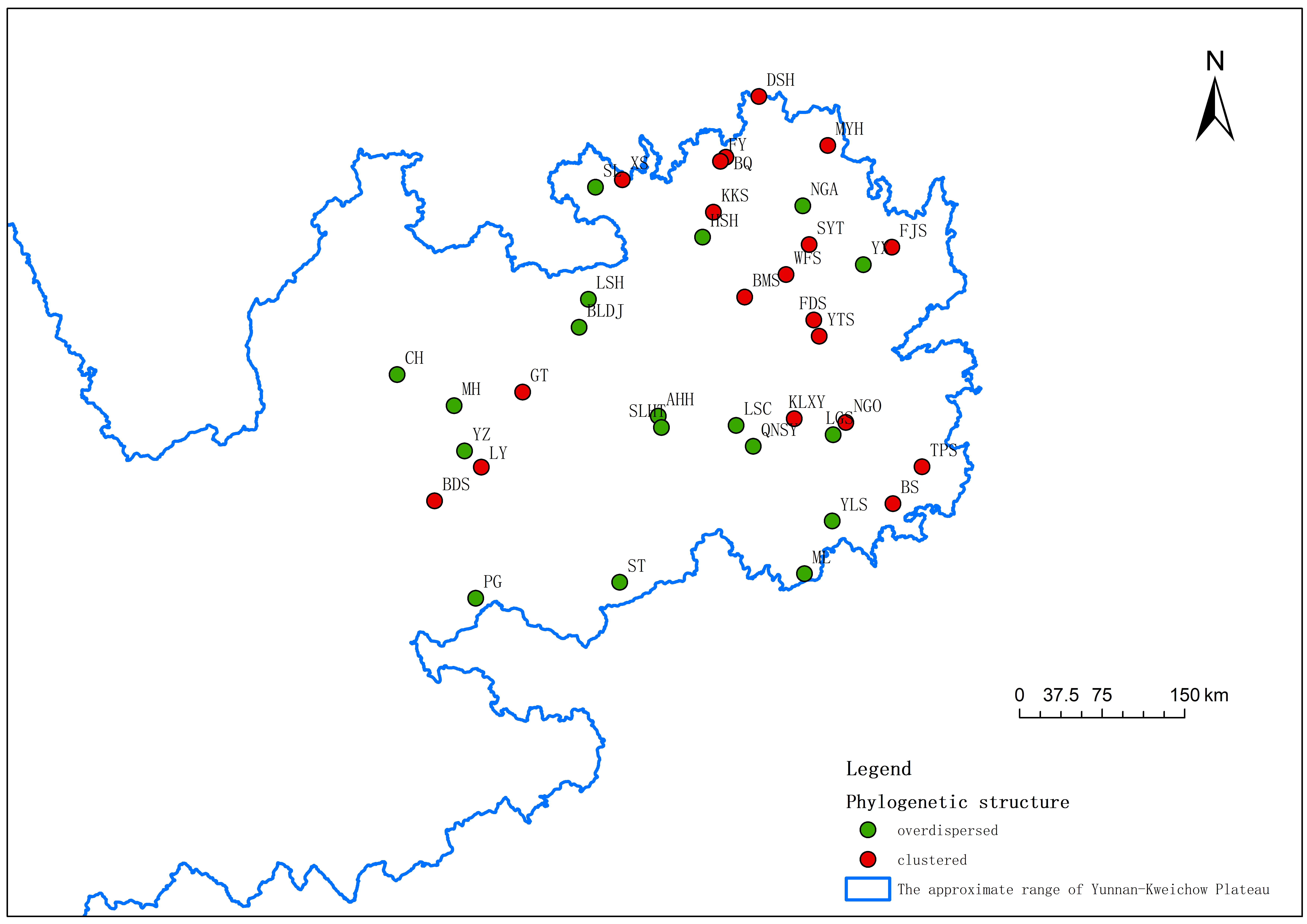

Supplement: Supplementary file 9 — Appendix S9. [file ECE3-13-e9735-s010.jpg]

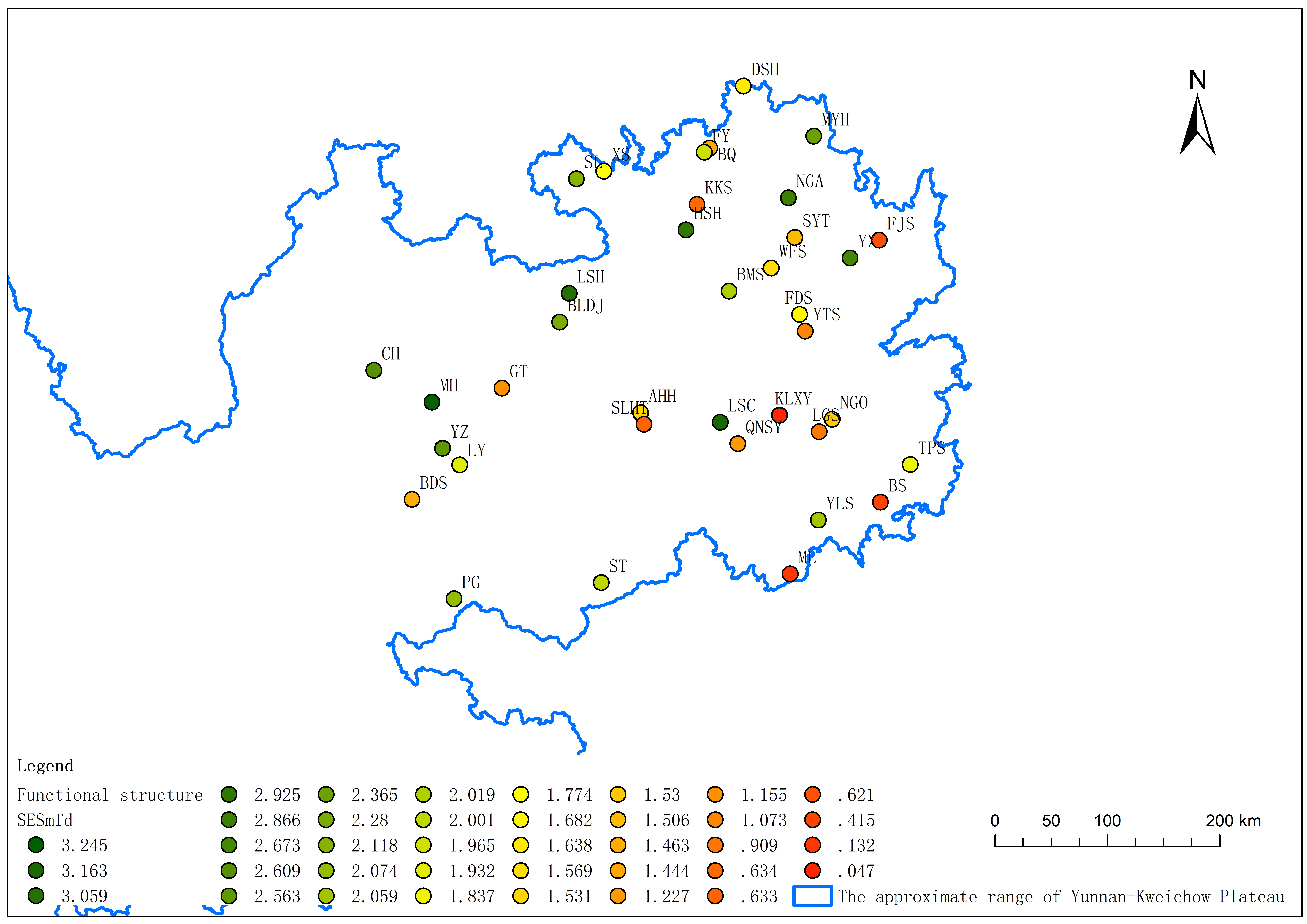

Supplement: Supplementary file 10 — Appendix S10. [file ECE3-13-e9735-s013.jpg]
