# Supplementary material for: The diversity of resident passerine bird in the East Yunnan‐Kweichow Plateau is closely related to plant species richness, vertical altitude difference and habitat area
Source: Ecol Evol. 2023 Jan 17;13(1):e9735. doi: 10.1002/ece3.9735 (PMC9843479; doi:10.1002/ece3.9735)
Supplement: Supplementary file 3 — Appendix S3. [file ECE3-13-e9735-s004.docx]

**Appendix S3** **List of 164 passerine resident birds in 37 sites of East Yunnan-Kweichow Plateau**

| **Species name** | **AHH** | **BDS** | **BS** | **BLDJ** | **BMS** | **BQ** | **CH** | **DSH** | **FJS** | **FDS** | **FY** | **GT** | **HSH** | **KLXY** | **KKS** | **LSC** | **LGS** | **LSH** | **LY** | **MYH** | **ML** | **MH** | **NGO** | **NGA** | **PG** | **QNSY** | **SLHT** | **SYT** | **ST** | **SL** | **TPS** | **WFS** | **XS** | **YX** | **YZ** | **YLS** | **YTS** |
| --- | --- | --- | --- | --- | --- | --- | --- | --- | --- | --- | --- | --- | --- | --- | --- | --- | --- | --- | --- | --- | --- | --- | --- | --- | --- | --- | --- | --- | --- | --- | --- | --- | --- | --- | --- | --- | --- |
| Zosterops_japonicus | √ |  |  |  |  |  |  |  |  |  |  |  |  |  |  |  |  |  | √ |  |  | √ | √ |  |  | √ | √ | √ |  |  | √ | √ |  |  |  | √ |  |
| Prinia_rufescens |  |  |  |  |  |  |  |  | √ |  |  |  |  |  |  |  |  |  |  |  |  |  |  |  | √ |  |  |  |  |  |  |  |  |  |  |  |  |
| Paradoxornis_zappeyi |  | √ |  |  |  |  | √ |  |  |  |  |  |  |  |  |  |  |  |  |  |  | √ |  |  |  |  |  |  |  |  |  |  |  |  |  |  |  |
| Acridotheres_cristatellus | √ | √ |  | √ | √ | √ | √ | √ | √ | √ | √ | √ | √ |  | √ | √ | √ | √ | √ | √ | √ | √ | √ | √ | √ | √ | √ | √ | √ | √ | √ | √ | √ | √ | √ | √ | √ |
| Chaimarrornis_leucocephalus | √ | √ |  |  | √ | √ | √ | √ | √ | √ | √ | √ |  |  | √ | √ | √ | √ | √ | √ | √ | √ |  |  | √ |  |  | √ |  | √ |  |  | √ |  |  |  | √ |
| Enicurus_leschenaulti |  |  | √ |  | √ | √ |  | √ | √ | √ | √ | √ |  |  | √ | √ | √ | √ |  |  | √ | √ | √ | √ | √ |  |  | √ | √ | √ | √ | √ | √ | √ | √ | √ | √ |
| Hodgsonius_phaenicuroides |  |  |  |  |  | √ |  | √ |  |  |  | √ |  |  | √ |  | √ |  |  |  |  |  |  |  |  |  |  |  |  |  |  |  |  |  |  |  | √ |
| Erpornis_zantholeuca |  |  |  |  |  |  |  |  |  |  |  |  |  |  | √ |  | √ |  |  |  | √ |  |  |  |  |  |  |  |  |  |  |  |  |  |  | √ |  |
| Brachypteryx_leucophrys |  |  |  |  |  |  |  |  | √ |  |  |  |  |  |  |  |  |  |  |  |  |  |  |  |  |  |  |  |  |  |  |  |  |  |  |  |  |
| Alophoixus_pallidus |  |  |  |  |  |  |  |  |  |  |  |  |  |  |  |  |  |  |  |  | √ |  |  |  |  |  |  |  |  |  |  |  |  |  |  |  |  |
| Pycnonotus_aurigaster |  |  |  |  |  |  |  |  |  |  |  |  |  |  |  |  |  |  | √ |  | √ |  |  |  | √ |  |  |  | √ |  |  |  |  |  | √ |  |  |
| Garrulax_albogularis |  |  |  |  |  |  |  | √ |  |  |  |  |  |  |  |  |  |  |  |  |  |  |  |  |  |  |  |  |  |  |  |  |  |  |  |  |  |
| Motacilla_alba | √ | √ | √ | √ | √ | √ | √ | √ | √ | √ | √ | √ | √ | √ | √ | √ | √ | √ | √ | √ | √ | √ | √ | √ | √ | √ | √ | √ | √ | √ | √ | √ | √ | √ | √ | √ | √ |
| Garrulax_sannio | √ | √ | √ | √ | √ | √ | √ | √ | √ | √ | √ | √ | √ | √ | √ | √ | √ | √ | √ | √ | √ | √ | √ | √ | √ | √ | √ | √ | √ | √ | √ | √ | √ | √ | √ | √ | √ |
| Corvus_torquatus |  | √ |  | √ | √ | √ | √ | √ | √ | √ | √ |  | √ |  | √ | √ | √ | √ |  | √ |  | √ |  | √ |  |  |  | √ |  | √ |  |  | √ | √ | √ | √ |  |
| Seicercus_affinis |  |  |  |  |  | √ |  |  | √ |  |  |  |  |  | √ |  |  |  |  |  |  | √ |  |  | √ |  |  |  |  |  |  |  |  |  |  |  |  |
| Yuhina_diademata |  | √ |  |  | √ | √ | √ | √ | √ | √ | √ | √ | √ |  | √ |  | √ | √ | √ | √ | √ | √ | √ |  |  |  |  |  |  | √ | √ | √ | √ |  | √ |  |  |
| Pycnonotus_sinensis | √ |  | √ |  |  | √ | √ | √ | √ |  |  |  |  | √ | √ |  | √ | √ | √ | √ | √ |  |  | √ |  | √ |  | √ |  | √ |  |  | √ | √ |  |  |  |
| Cinclidium_leucurum |  | √ |  |  |  | √ |  | √ | √ | √ |  |  | √ |  | √ |  |  |  |  | √ |  |  |  |  | √ |  |  |  |  |  |  |  |  |  |  |  | √ |
| Lonchura_striata | √ | √ | √ | √ | √ | √ |  | √ | √ | √ | √ | √ |  |  |  | √ | √ | √ | √ | √ | √ | √ | √ | √ | √ | √ |  | √ | √ | √ | √ | √ | √ | √ | √ | √ | √ |
| Enicurus_maculatus |  |  |  |  |  |  |  |  |  |  |  |  |  |  |  |  | √ |  |  |  |  |  |  |  |  |  |  |  |  |  |  |  |  |  |  |  |  |
| Spelaeornis_troglodytoides |  |  |  |  |  |  |  |  | √ |  |  |  |  |  |  |  |  |  |  |  |  |  |  |  |  |  |  |  |  |  |  |  |  |  |  |  |  |
| Lonchura_punctulata | √ |  |  |  |  |  |  |  |  |  |  |  |  | √ |  |  |  |  |  |  | √ |  |  | √ | √ |  |  |  | √ |  |  |  |  |  | √ | √ |  |
| Pomatorhinus_mcclellandi | √ | √ | √ | √ |  | √ | √ | √ | √ |  |  | √ | √ |  | √ | √ |  | √ | √ | √ |  | √ |  | √ | √ |  |  | √ | √ | √ |  | √ | √ | √ | √ | √ |  |
| Turdus_mupinensis |  | √ |  | √ |  |  | √ | √ |  | √ |  | √ |  |  |  |  |  |  |  | √ | √ |  |  |  | √ |  |  |  |  | √ |  |  | √ |  | √ |  |  |
| Phoenicurus_auroreus | √ | √ | √ | √ | √ | √ | √ | √ | √ | √ | √ | √ | √ | √ | √ | √ | √ | √ | √ | √ | √ | √ |  | √ | √ | √ |  | √ | √ | √ | √ | √ | √ | √ | √ | √ | √ |
| Serinus_thibetanus |  |  |  |  |  |  |  |  |  |  |  |  |  | √ |  |  |  |  |  |  |  |  |  |  |  |  |  |  |  |  |  |  |  |  |  |  |  |
| Aethopyga_christinae | √ |  | √ |  |  |  |  |  | √ |  |  |  |  |  |  |  |  |  |  | √ | √ |  |  |  |  |  |  |  |  | √ |  |  | √ | √ |  | √ |  |
| Garrulax_elliotii |  | √ |  | √ |  | √ | √ | √ | √ |  |  | √ | √ |  |  |  |  |  | √ |  |  | √ |  |  |  |  |  |  |  | √ |  |  |  |  | √ |  |  |
| Chloropsis_hardwickii | √ |  |  |  |  |  |  |  | √ |  |  |  |  |  |  | √ | √ |  |  |  | √ |  | √ |  |  |  |  |  |  |  | √ |  |  |  |  |  |  |
| Pericrocotus_flammeus |  | √ |  |  |  |  |  |  |  |  |  |  |  |  |  |  | √ |  | √ |  | √ |  |  |  |  |  |  |  | √ |  |  |  |  |  |  |  |  |
| Prinia_inornata | √ | √ | √ |  |  | √ | √ | √ | √ |  |  |  |  | √ |  | √ | √ | √ | √ | √ | √ |  |  |  | √ | √ | √ | √ | √ | √ |  | √ | √ |  | √ | √ |  |
| Dicaeum_concolor |  |  |  |  |  |  |  | √ |  |  |  |  |  |  |  |  |  |  |  | √ | √ |  | √ |  | √ |  |  |  | √ | √ | √ |  | √ |  |  | √ |  |
| Coracina_macei |  |  |  |  |  |  |  |  |  |  |  |  |  |  |  |  |  |  |  | √ | √ |  |  |  | √ |  |  |  |  |  |  |  |  |  |  |  |  |
| Parus_major | √ | √ | √ | √ | √ | √ | √ | √ | √ | √ | √ | √ | √ | √ | √ | √ | √ | √ | √ | √ | √ | √ | √ | √ | √ | √ | √ | √ | √ | √ | √ | √ | √ | √ | √ | √ | √ |
| Corvus_macrorhynchos |  | √ |  | √ | √ | √ | √ | √ | √ |  | √ |  | √ |  | √ | √ | √ | √ | √ | √ | √ | √ | √ | √ | √ |  |  |  |  | √ | √ |  | √ |  | √ | √ | √ |
| Regulus_regulus |  | √ |  |  |  |  | √ | √ |  |  |  |  | √ |  |  |  |  |  |  |  |  | √ |  |  |  |  |  |  |  | √ |  |  |  |  | √ |  |  |
| Zoothera_mollissima |  |  |  |  |  |  |  |  |  |  |  |  |  |  |  |  |  |  |  |  |  |  |  |  |  |  |  |  |  | √ |  |  |  |  |  |  |  |
| Sitta_yunnanensis |  |  |  |  |  |  | √ |  |  |  |  |  |  |  |  |  |  |  |  |  |  | √ |  |  |  |  |  |  |  |  |  |  |  |  | √ |  |  |
| Paradoxornis_guttaticollis | √ | √ |  |  |  | √ | √ |  | √ |  |  |  | √ |  | √ | √ |  |  |  | √ | √ | √ |  |  | √ |  |  |  | √ |  | √ |  |  |  | √ | √ |  |
| Pericrocotus_brevirostris | √ |  |  |  |  |  |  |  | √ | √ |  |  |  |  | √ |  | √ |  | √ |  | √ |  | √ |  | √ |  |  |  |  | √ |  |  |  | √ | √ |  |  |
| Melophus_lathami |  | √ |  | √ |  |  |  | √ | √ | √ |  |  |  |  |  | √ | √ | √ | √ | √ | √ |  | √ |  | √ |  |  |  | √ | √ | √ |  | √ | √ | √ | √ |  |
| Tephrodornis_gularis |  |  |  |  |  |  |  |  |  |  |  |  |  |  |  |  |  |  |  |  |  |  |  |  | √ |  |  |  | √ |  |  |  |  |  |  |  |  |
| Alcippe_brunnea |  |  |  |  |  |  |  | √ |  |  |  |  |  |  | √ |  |  |  |  | √ |  |  |  |  |  |  |  |  |  |  |  |  | √ |  |  |  |  |
| Cinclus_pallasii | √ | √ |  |  | √ | √ |  | √ | √ | √ | √ |  |  |  | √ | √ | √ | √ | √ | √ | √ |  | √ | √ |  |  |  |  | √ | √ | √ |  | √ | √ | √ | √ | √ |
| Alcippe_manipurensis |  | √ |  |  |  | √ | √ | √ | √ |  |  | √ |  |  | √ |  | √ |  |  |  |  |  |  |  |  |  |  |  |  |  |  |  |  | √ | √ |  |  |
| Alcippe_dubia | √ | √ |  |  | √ | √ | √ | √ | √ | √ | √ | √ | √ | √ | √ | √ | √ | √ | √ | √ | √ |  | √ | √ | √ |  | √ | √ |  | √ | √ |  | √ | √ | √ | √ | √ |
| Garrulax_maesi |  |  |  |  |  |  |  |  |  |  |  |  |  |  | √ |  | √ |  |  |  |  |  | √ |  |  |  |  |  |  |  | √ |  | √ |  |  | √ |  |
| Enicurus_immaculatus |  | √ |  |  |  |  |  |  |  |  |  |  |  |  |  |  |  |  |  | √ |  |  |  |  |  |  |  |  |  |  |  |  |  |  |  |  |  |
| Pycnonotus_melanicterus |  |  |  |  |  |  |  |  |  |  |  |  |  |  |  |  |  |  |  |  |  |  |  |  |  |  |  |  | √ |  |  |  |  |  |  |  |  |
| Prinia_atrogularis |  |  | √ |  |  |  |  |  |  |  |  |  |  |  |  |  | √ |  | √ |  | √ |  |  |  |  |  |  |  | √ |  |  |  |  |  |  | √ |  |
| Saxicola_torquatus | √ | √ | √ | √ | √ | √ | √ | √ | √ |  | √ | √ |  | √ | √ | √ | √ | √ | √ | √ | √ | √ | √ | √ | √ | √ |  | √ | √ | √ | √ |  | √ | √ | √ | √ | √ |
| Garrulax_chinensis |  |  |  |  |  |  |  |  |  |  |  |  |  |  |  |  |  |  |  |  |  |  |  |  |  |  |  |  |  | √ |  |  | √ |  |  |  |  |
| Yuhina_nigrimenta |  |  | √ |  | √ | √ |  | √ | √ | √ | √ |  |  |  | √ |  | √ |  | √ | √ | √ |  | √ |  |  |  |  | √ | √ | √ | √ | √ | √ | √ |  | √ | √ |
| Garrulax_perspicillatus |  |  |  |  | √ |  |  | √ | √ | √ | √ |  |  |  | √ | √ |  |  | √ | √ |  |  |  |  |  |  |  | √ |  |  |  |  | √ | √ |  |  |  |
| Garrulax_pectoralis |  |  |  |  | √ | √ |  |  |  | √ | √ |  |  |  | √ |  |  |  |  |  | √ |  |  |  |  |  |  |  |  |  |  |  |  |  |  |  | √ |
| Aegithalos_iouschistos |  | √ |  |  |  |  | √ |  |  |  |  |  |  |  |  |  |  |  |  |  |  | √ |  |  |  |  |  |  |  |  |  |  |  |  | √ |  | √ |
| Pheucticus_melanocephalus |  | √ |  |  |  |  | √ |  |  |  |  |  | √ | √ |  |  |  |  |  |  |  | √ |  |  | √ |  |  |  |  |  |  |  |  |  | √ |  |  |
| Heterophasia_desgodinsi |  |  |  |  | √ | √ |  | √ | √ | √ | √ | √ |  |  | √ |  |  |  | √ | √ |  |  |  | √ |  |  |  |  | √ |  |  |  |  |  | √ |  |  |
| Turdus_dissimilis | √ | √ |  |  |  |  | √ |  |  |  |  | √ |  |  |  |  |  | √ | √ |  |  |  |  |  | √ | √ | √ | √ | √ |  |  | √ |  | √ | √ |  |  |
| Aethopyga_saturata |  |  |  |  |  |  |  |  |  |  |  |  |  |  |  |  |  |  |  |  |  |  |  |  | √ |  |  |  | √ |  |  |  |  |  | √ |  |  |
| Pteruthius_flaviscapis |  |  |  |  |  |  |  |  |  |  |  |  |  |  |  |  | √ |  |  |  |  |  |  |  | √ |  |  |  | √ | √ |  |  |  |  |  |  |  |
| Timalia_pileata |  |  |  |  |  |  |  |  |  |  |  |  |  |  |  |  |  |  |  |  | √ |  | √ |  |  |  |  |  | √ |  | √ |  |  |  |  | √ |  |
| Pycnonotus_jocosus |  |  |  |  |  |  |  |  |  |  |  |  |  |  |  |  |  |  | √ |  | √ |  |  |  | √ |  |  |  | √ |  |  |  |  |  |  |  |  |
| Stachyris_ruficeps | √ | √ | √ | √ | √ | √ |  | √ | √ | √ | √ | √ |  | √ | √ | √ | √ | √ | √ | √ | √ |  | √ | √ | √ | √ | √ | √ | √ | √ | √ | √ | √ | √ | √ | √ | √ |
| Aegithalos_concinnus | √ | √ | √ |  | √ | √ | √ | √ | √ | √ | √ | √ | √ | √ | √ | √ | √ | √ | √ | √ | √ | √ | √ | √ | √ | √ | √ | √ | √ | √ | √ | √ | √ | √ | √ | √ | √ |
| Rhyacornis_fuliginosa | √ | √ | √ | √ | √ | √ | √ | √ | √ | √ | √ | √ | √ | √ | √ | √ | √ | √ | √ | √ | √ | √ | √ | √ | √ | √ | √ | √ | √ | √ | √ | √ | √ | √ | √ | √ | √ |
| Minla_ignotincta |  |  |  |  |  | √ |  | √ |  |  |  |  |  |  | √ |  | √ |  |  | √ |  |  | √ |  |  |  |  |  |  | √ |  |  | √ |  |  |  |  |
| Garrulax_milnei |  |  |  |  |  | √ |  | √ |  |  | √ |  |  |  | √ |  |  |  |  |  |  |  |  |  |  |  |  |  |  | √ |  |  | √ |  |  |  |  |
| Dicaeum_ignipectus |  |  | √ |  |  |  |  |  | √ |  |  |  |  |  |  | √ |  |  | √ |  |  |  |  |  |  |  |  |  | √ |  |  |  |  |  | √ |  | √ |
| Urocissa_erythrorhyncha | √ | √ | √ | √ | √ | √ | √ | √ | √ | √ | √ | √ | √ | √ | √ | √ | √ | √ | √ | √ | √ | √ | √ | √ | √ | √ | √ | √ | √ | √ | √ | √ | √ | √ | √ | √ | √ |
| Leiothrix_lutea | √ | √ | √ | √ | √ | √ | √ | √ | √ | √ | √ | √ |  |  | √ | √ | √ | √ | √ | √ | √ | √ | √ | √ | √ | √ |  | √ | √ | √ | √ | √ | √ | √ | √ | √ | √ |
| Garrulax_canorus | √ | √ | √ | √ | √ | √ | √ | √ | √ | √ | √ | √ |  | √ | √ | √ | √ | √ |  | √ | √ | √ | √ | √ | √ |  |  | √ | √ | √ | √ | √ | √ | √ | √ | √ |  |
| Prinia_flaviventris |  |  |  |  |  |  |  |  | √ |  |  |  |  |  |  |  | √ |  |  |  | √ |  |  |  | √ |  |  |  | √ |  |  |  |  |  |  | √ |  |
| Parus_venustulus |  |  |  | √ | √ | √ | √ | √ | √ | √ | √ |  |  |  | √ | √ | √ |  | √ | √ | √ |  | √ | √ |  |  |  |  |  | √ |  |  | √ | √ |  | √ | √ |
| Cettia_acanthizoides |  | √ |  |  |  |  |  | √ |  |  |  | √ | √ |  | √ |  | √ |  |  | √ |  | √ |  |  |  |  |  |  |  | √ |  |  |  |  |  |  | √ |
| Emberiza_elegans | √ | √ | √ | √ |  | √ | √ | √ | √ |  |  | √ | √ |  | √ | √ | √ | √ | √ | √ | √ | √ |  | √ |  |  |  | √ |  | √ |  | √ | √ | √ | √ | √ |  |
| Parus_spilonotus |  |  |  |  |  |  |  |  |  |  |  |  |  |  |  |  | √ |  |  |  | √ |  |  |  |  |  |  |  | √ |  |  |  |  |  |  | √ |  |
| Sylviparus_modestus |  |  |  |  |  | √ |  |  | √ |  |  |  |  |  |  |  | √ |  |  |  |  |  |  |  |  |  |  |  |  |  |  |  |  |  |  |  |  |
| Pycnonotus_xanthorrhous | √ | √ | √ | √ | √ | √ | √ | √ | √ | √ | √ | √ | √ | √ | √ | √ | √ | √ | √ | √ | √ | √ | √ | √ | √ | √ | √ | √ | √ | √ | √ | √ | √ | √ | √ | √ | √ |
| Phylloscopus_cantator |  |  |  |  |  |  |  | √ |  |  |  |  |  |  |  |  |  |  |  |  |  |  |  |  |  |  |  |  |  | √ |  |  |  | √ |  |  |  |
| Enicurus_schistaceus |  |  |  | √ |  | √ |  | √ | √ | √ |  |  |  |  | √ |  | √ | √ | √ | √ | √ |  |  | √ |  |  |  |  | √ | √ | √ |  | √ | √ | √ | √ | √ |
| Turdus_boulboul |  |  |  |  |  |  |  | √ | √ | √ |  |  |  |  | √ |  |  |  |  |  | √ |  |  |  | √ |  |  |  |  | √ |  |  |  |  |  |  |  |
| Garrulax_cineraceus |  |  |  |  | √ |  |  | √ | √ |  | √ |  |  |  | √ |  |  |  |  | √ | √ |  |  |  | √ |  |  |  | √ | √ |  |  | √ |  |  |  |  |
| Zosterops_palpebrosus |  |  |  |  |  |  |  |  | √ | √ |  | √ |  |  |  |  |  |  |  |  | √ |  |  |  | √ |  |  |  | √ |  |  |  | √ |  | √ | √ | √ |
| Seicercus_tephrocephalus |  | √ |  |  | √ |  |  | √ | √ |  | √ |  | √ |  | √ |  | √ | √ |  |  |  |  |  |  |  |  |  |  |  | √ |  |  | √ |  | √ |  |  |
| Pericrocotus_solaris |  |  | √ |  |  | √ |  |  | √ | √ |  |  |  |  |  |  | √ |  |  |  | √ |  | √ |  |  | √ |  |  |  | √ | √ |  |  |  |  | √ |  |
| Paradoxornis_alphonsianus | √ | √ |  | √ |  | √ |  |  |  |  |  |  |  |  | √ |  |  | √ |  |  |  |  |  | √ | √ | √ | √ |  |  |  |  |  |  |  | √ |  |  |
| Alcippe_morrisonia | √ | √ | √ | √ | √ | √ |  | √ | √ | √ | √ | √ |  |  | √ | √ | √ | √ | √ | √ | √ |  | √ | √ | √ |  |  | √ | √ | √ | √ | √ | √ | √ |  | √ | √ |
| Saxicola_ferreus | √ | √ | √ | √ | √ | √ |  | √ | √ | √ | √ | √ |  |  | √ | √ | √ | √ | √ | √ | √ |  | √ | √ | √ |  |  | √ | √ |  | √ | √ | √ | √ | √ | √ | √ |
| Emberiza_godlewskii | √ | √ |  | √ |  | √ | √ | √ |  |  |  | √ | √ |  | √ | √ |  | √ | √ | √ |  | √ |  | √ | √ | √ |  |  | √ | √ |  | √ | √ |  | √ |  |  |
| Dendrocitta_formosae |  | √ |  |  | √ | √ |  | √ | √ | √ | √ | √ |  |  | √ | √ | √ | √ | √ | √ | √ |  |  | √ |  |  |  |  |  | √ |  |  | √ | √ |  | √ |  |
| Turdus_rubrocanus |  |  |  |  |  |  |  | √ | √ |  |  |  |  |  |  |  |  |  |  | √ | √ |  |  |  |  |  |  |  |  |  |  |  |  |  |  |  |  |
| Pyrrhula_erythaca |  |  |  |  |  |  |  |  | √ |  |  |  |  |  |  |  |  |  |  |  |  |  |  |  |  |  |  |  |  |  |  |  |  |  |  |  |  |
| Sturnus_malabaricus |  |  |  |  |  |  |  |  |  |  |  |  |  |  |  |  |  |  |  |  |  |  |  |  | √ |  |  |  | √ |  |  |  |  |  |  |  |  |
| Paradoxornis_gularis |  |  |  |  | √ | √ |  | √ | √ | √ | √ |  |  |  | √ |  | √ |  |  | √ | √ |  |  |  |  |  |  | √ | √ | √ |  |  | √ |  |  |  | √ |
| Cyanopica_cyanus | √ |  |  |  |  |  |  |  |  |  |  |  |  |  |  |  |  |  |  |  |  |  |  |  |  |  |  |  |  |  |  |  |  |  |  |  |  |
| Prinia_hodgsonii |  |  |  |  |  |  |  | √ |  |  |  |  |  |  |  |  |  |  |  |  | √ |  |  |  | √ |  |  |  | √ |  |  |  |  |  | √ |  |  |
| Artamus_fuscus |  |  |  |  |  |  |  |  |  |  |  |  |  |  |  |  |  |  |  |  | √ |  |  |  |  |  |  |  |  |  |  |  |  |  |  |  |  |
| Cephalopyrus_flammiceps |  |  |  |  |  |  |  |  |  |  |  |  |  |  |  |  |  |  |  |  |  | √ |  |  |  |  |  |  |  |  |  |  |  |  |  |  |  |
| Carduelis_sinica | √ | √ |  | √ | √ | √ | √ | √ | √ | √ | √ | √ |  | √ | √ | √ | √ | √ | √ | √ | √ | √ | √ | √ | √ | √ | √ | √ | √ | √ | √ | √ | √ | √ | √ | √ | √ |
| Tesia_olivea |  |  |  |  |  |  |  |  |  |  |  |  |  |  |  |  | √ |  |  |  |  |  |  |  |  |  |  |  |  |  |  |  |  |  |  |  |  |
| Paradoxornis_verreauxi |  |  |  |  |  | √ |  |  | √ |  |  |  |  |  | √ |  |  |  |  |  |  |  |  |  |  |  |  |  |  |  |  |  |  |  |  |  |  |
| Cisticola_exilis |  |  |  |  |  |  |  |  |  |  |  |  |  |  |  |  |  |  |  |  |  |  |  |  |  |  |  |  |  |  |  |  |  |  |  | √ |  |
| Alcippe_chrysotis |  |  |  |  | √ | √ |  | √ | √ | √ | √ |  |  |  | √ |  | √ |  |  | √ |  |  |  |  |  |  |  |  |  |  |  |  |  |  |  |  |  |
| Carpodacus_vinaceus |  | √ |  |  | √ | √ | √ | √ | √ | √ | √ | √ |  |  | √ |  |  |  | √ |  |  | √ |  |  |  |  |  |  |  | √ |  |  |  |  |  |  |  |
| Minla_cyanouroptera |  | √ |  |  |  | √ |  | √ | √ |  |  | √ |  | √ | √ |  | √ |  | √ | √ |  |  |  |  | √ | √ |  |  |  | √ |  |  | √ |  | √ |  |  |
| Brachypteryx_montana |  |  |  |  |  |  |  |  |  |  |  |  |  |  |  |  |  |  |  |  | √ |  |  |  |  |  |  |  |  |  |  |  |  |  |  |  |  |
| Phoenicurus_frontalis |  | √ |  |  |  | √ | √ | √ |  |  |  | √ | √ |  | √ |  |  |  |  | √ |  |  |  |  |  |  |  |  |  | √ |  |  |  |  | √ |  |  |
| Aethopyga_gouldiae |  | √ |  |  | √ | √ |  | √ | √ | √ | √ | √ | √ |  | √ | √ | √ | √ | √ | √ | √ | √ | √ |  | √ |  |  | √ |  | √ | √ |  | √ | √ | √ | √ |  |
| Monticola_solitarius |  | √ |  |  |  |  | √ | √ | √ |  |  |  |  |  |  |  |  | √ | √ | √ | √ | √ |  |  | √ |  |  |  | √ | √ |  |  | √ | √ | √ | √ |  |
| Lanius_collurioides |  |  |  |  |  |  |  |  |  |  |  |  |  |  |  |  |  |  |  |  |  |  |  |  |  |  |  |  | √ |  |  |  |  |  |  |  |  |
| Brachypteryx_stellata |  |  |  | √ |  |  | √ |  |  |  |  |  |  |  |  |  |  |  |  |  |  |  |  |  | √ |  |  |  |  |  |  |  |  |  |  |  |  |
| Hemixos_castanonotus | √ |  |  |  |  |  |  |  | √ |  |  |  |  |  | √ |  | √ |  | √ |  | √ |  | √ |  |  |  |  |  | √ |  | √ |  |  | √ |  | √ |  |
| Yuhina_castaniceps | √ |  | √ |  |  | √ |  | √ | √ | √ |  |  |  |  | √ |  | √ | √ | √ | √ | √ |  | √ | √ | √ | √ |  | √ | √ | √ | √ |  | √ | √ | √ | √ | √ |
| Monticola_rufiventris |  | √ |  |  |  | √ |  | √ | √ |  | √ |  |  |  | √ | √ | √ |  | √ | √ |  |  | √ |  |  |  |  |  | √ |  | √ |  |  |  | √ |  |  |
| Tesia_castaneocoronata |  |  |  |  |  |  | √ |  |  |  |  |  |  |  |  |  |  |  |  |  |  | √ |  |  |  |  |  |  |  | √ |  |  | √ |  |  |  |  |
| Spizixos_semitorques | √ | √ | √ | √ | √ | √ | √ | √ | √ | √ | √ | √ | √ | √ | √ | √ | √ | √ | √ | √ | √ | √ | √ | √ | √ | √ | √ | √ | √ | √ | √ | √ | √ | √ | √ | √ | √ |
| Parus_monticolus | √ | √ |  | √ | √ | √ | √ | √ | √ | √ | √ | √ | √ | √ | √ | √ | √ | √ | √ | √ |  | √ | √ | √ | √ |  |  |  |  | √ | √ | √ | √ | √ | √ | √ | √ |
| Hypsipetes_mcclellandii | √ | √ |  | √ | √ | √ |  | √ | √ | √ | √ | √ |  |  | √ | √ | √ | √ | √ | √ | √ |  | √ | √ | √ | √ |  | √ | √ | √ | √ |  | √ | √ | √ | √ |  |
| Passer_montanus | √ | √ | √ | √ | √ | √ | √ | √ | √ | √ | √ | √ | √ | √ | √ | √ | √ | √ | √ | √ | √ | √ | √ | √ | √ | √ | √ | √ | √ | √ | √ | √ | √ | √ | √ | √ |  |
| Babax_lanceolatus | √ | √ |  | √ | √ | √ | √ | √ | √ | √ | √ | √ | √ |  | √ | √ | √ | √ | √ | √ | √ | √ | √ | √ | √ |  |  | √ | √ | √ | √ | √ | √ | √ | √ | √ | √ |
| Parus_ater |  |  |  |  |  |  | √ | √ |  |  |  |  |  |  |  |  |  |  |  |  |  | √ |  |  |  |  |  |  |  |  |  |  |  |  |  |  |  |
| Sitta_europaea |  | √ |  |  | √ |  | √ | √ | √ | √ |  |  | √ |  |  | √ |  |  |  | √ | √ | √ | √ |  |  |  |  | √ |  | √ | √ |  |  | √ | √ | √ |  |
| Cettia_fortipes | √ | √ | √ | √ | √ | √ | √ | √ | √ | √ | √ | √ | √ | √ | √ | √ | √ | √ | √ | √ | √ | √ | √ | √ |  | √ | √ | √ | √ | √ | √ | √ | √ | √ | √ | √ | √ |
| Copsychus_saularis | √ | √ | √ | √ | √ | √ | √ | √ | √ | √ | √ | √ | √ | √ | √ | √ |  | √ | √ | √ | √ | √ | √ | √ | √ | √ | √ | √ | √ | √ | √ | √ | √ | √ | √ | √ | √ |
| Sitta_frontalis |  |  |  |  |  |  |  |  |  |  |  |  |  |  |  |  |  |  |  |  |  |  |  |  |  |  |  |  | √ |  |  |  |  |  |  |  |  |
| Emberiza_cioides | √ | √ |  | √ | √ | √ | √ | √ | √ | √ | √ | √ | √ |  | √ | √ | √ | √ | √ | √ | √ | √ | √ | √ | √ | √ |  | √ | √ | √ | √ | √ | √ | √ | √ | √ |  |
| Prinia_crinigera | √ | √ |  |  |  |  | √ |  | √ |  |  | √ |  |  | √ |  | √ |  | √ | √ |  |  |  |  | √ |  |  | √ | √ |  |  |  | √ |  | √ | √ |  |
| Anthus_sylvanus |  | √ |  |  |  |  |  |  |  |  |  |  |  |  |  |  |  |  |  | √ |  |  |  |  | √ |  |  |  |  |  |  |  |  |  | √ | √ |  |
| Passer_rutilans | √ | √ | √ | √ | √ | √ | √ | √ | √ | √ | √ | √ | √ |  | √ | √ | √ | √ | √ | √ | √ | √ | √ | √ | √ | √ | √ | √ | √ | √ | √ | √ | √ | √ | √ | √ | √ |
| Sturnus_sericeus | √ |  |  |  |  |  | √ | √ | √ | √ |  |  |  |  |  |  | √ |  | √ | √ | √ |  |  |  |  |  | √ |  |  | √ |  |  |  |  |  |  |  |
| Garrulus_glandarius | √ | √ |  |  | √ | √ | √ | √ | √ | √ | √ | √ | √ |  | √ | √ | √ | √ | √ | √ |  | √ |  | √ | √ |  |  | √ | √ | √ |  | √ | √ | √ | √ | √ | √ |
| Arachnothera_magna |  |  |  |  |  |  |  |  |  |  |  |  |  |  |  |  |  |  |  |  |  |  |  |  | √ |  |  |  |  |  |  |  |  |  |  |  |  |
| Turdus_merula | √ | √ |  | √ | √ |  | √ | √ | √ | √ | √ | √ |  | √ |  | √ | √ |  | √ | √ | √ | √ | √ | √ | √ | √ | √ | √ | √ | √ | √ | √ | √ | √ | √ | √ | √ |
| Pica_pica | √ | √ | √ | √ | √ | √ | √ | √ | √ | √ | √ | √ | √ |  | √ | √ |  | √ | √ | √ | √ | √ | √ | √ | √ | √ |  | √ | √ | √ | √ | √ | √ | √ | √ | √ |  |
| Ficedula_westermanni |  |  |  |  |  |  |  |  |  |  |  |  |  |  | √ |  |  |  |  |  |  |  |  |  |  |  |  |  | √ |  |  |  |  |  |  |  | √ |
| Garrulax_monileger |  |  |  |  |  |  |  |  |  |  |  |  |  |  |  |  |  |  |  |  | √ |  |  |  |  |  |  |  |  |  |  |  |  |  |  |  |  |
| Pnoepyga_pusilla |  |  |  |  |  | √ |  | √ |  |  |  |  |  |  | √ |  | √ |  |  | √ | √ |  |  |  |  |  |  |  |  | √ |  |  | √ |  |  |  |  |
| Niltava_macgrigoriae |  |  |  |  |  |  |  |  |  |  |  |  |  |  |  |  | √ |  |  |  | √ |  |  |  |  |  |  |  |  |  |  |  |  |  |  |  |  |
| Enicurus_scouleri |  | √ | √ |  |  | √ |  | √ | √ |  |  | √ |  |  | √ |  | √ | √ |  | √ | √ |  |  | √ |  |  |  |  |  | √ | √ |  | √ |  |  | √ |  |
| Alauda_gulgula | √ | √ |  |  |  | √ | √ | √ | √ |  |  | √ |  |  | √ | √ | √ | √ | √ | √ |  | √ |  |  | √ |  |  |  |  |  |  |  |  |  | √ | √ |  |
| Corvus_corone |  |  |  |  |  |  | √ |  |  |  |  |  | √ |  |  |  |  |  |  |  |  | √ |  |  |  |  |  |  | √ | √ |  |  | √ | √ |  |  |  |
| Nucifraga_caryocatactes |  |  |  |  |  |  | √ |  |  |  |  |  |  |  |  |  |  |  |  |  |  | √ |  |  |  |  |  |  |  |  |  |  |  |  |  |  |  |
| Garrulax_ocellatus |  |  |  |  |  | √ |  | √ |  |  |  |  |  |  |  |  |  |  |  |  |  |  |  |  |  |  |  |  |  |  |  |  |  |  |  |  |  |
| Cettia_flavolivacea |  |  |  |  |  |  |  |  | √ |  |  |  |  |  |  |  |  |  |  |  |  |  |  |  |  |  |  |  |  |  |  |  |  |  |  |  |  |
| Leiothrix_argentauris |  |  |  |  |  |  |  |  |  |  |  |  |  |  |  |  |  |  |  |  |  |  | √ |  |  |  |  |  | √ |  |  |  |  |  |  |  |  |
| Orthotomus_sutorius |  |  | √ |  |  |  |  |  |  |  |  |  |  |  |  |  |  |  | √ |  | √ |  | √ |  | √ | √ |  |  | √ |  | √ |  | √ |  | √ | √ | √ |
| Psarisomus_dalhousiae |  |  |  |  |  |  |  |  |  |  |  |  |  |  |  |  |  |  |  |  | √ |  |  |  |  |  |  |  | √ |  |  |  |  |  |  |  |  |
| Megalurus_palustris |  |  |  |  |  |  |  |  |  |  |  |  |  |  |  |  |  |  |  |  |  |  |  |  |  |  |  |  | √ |  |  |  |  |  |  |  |  |
| Phoenicurus_ochruros |  |  |  |  |  |  |  |  |  |  |  |  |  |  |  |  |  |  |  |  |  |  |  |  |  |  |  |  |  | √ |  |  | √ |  |  |  |  |
| Graminicola_bengalensis |  |  |  |  |  |  |  |  |  |  |  |  |  |  |  |  |  |  |  |  | √ |  |  |  |  |  |  |  |  |  |  |  |  |  |  |  |  |
| Myophonus_caeruleus | √ | √ |  | √ | √ | √ | √ | √ | √ | √ | √ | √ | √ |  | √ | √ | √ | √ | √ | √ | √ | √ |  | √ | √ | √ |  | √ | √ | √ |  | √ | √ | √ | √ |  | √ |
| Lanius_schach | √ | √ | √ | √ | √ | √ | √ | √ | √ | √ | √ | √ | √ | √ | √ | √ | √ | √ | √ | √ | √ | √ | √ | √ | √ | √ | √ | √ | √ | √ | √ | √ | √ | √ | √ | √ | √ |
| Niltava_davidi |  |  |  |  |  |  |  | √ |  |  | √ |  |  |  | √ |  |  |  |  |  |  |  |  |  |  |  |  |  |  | √ |  |  |  |  |  |  |  |
| Niltava_sundara |  | √ |  |  |  |  | √ |  |  |  |  | √ | √ |  | √ |  |  |  |  |  | √ | √ |  |  |  |  |  |  |  | √ |  |  | √ |  | √ |  |  |
| Bradypterus_luteoventris |  | √ |  |  |  | √ |  | √ | √ |  |  |  |  |  | √ |  | √ | √ |  |  |  |  | √ |  |  |  |  |  |  |  | √ |  | √ |  |  | √ |  |
| Pomatorhinus_ruficollis | √ | √ | √ | √ | √ | √ | √ | √ | √ | √ | √ | √ | √ |  | √ | √ | √ | √ | √ | √ | √ | √ | √ | √ | √ |  |  | √ | √ | √ | √ | √ | √ | √ | √ | √ | √ |
| Abroscopus_albogularis | √ |  |  | √ | √ | √ |  | √ | √ | √ |  | √ |  |  | √ |  |  |  | √ | √ |  |  |  | √ | √ | √ | √ | √ |  |  |  | √ | √ | √ |  |  |  |
| Cisticola_juncidis |  | √ |  |  |  |  | √ |  |  |  |  |  |  |  |  |  |  |  | √ |  | √ | √ |  |  |  |  |  |  |  | √ |  |  | √ |  |  | √ |  |
| Alcippe_ruficapilla |  | √ |  |  |  |  | √ |  |  |  |  | √ |  |  |  |  |  |  | √ |  |  |  |  |  |  |  |  |  |  | √ |  |  |  |  |  |  |  |
| Paradoxornis_webbianus |  | √ | √ | √ | √ | √ | √ | √ | √ | √ | √ |  |  | √ | √ | √ | √ |  | √ | √ | √ | √ | √ | √ | √ | √ |  | √ | √ | √ | √ | √ | √ | √ |  | √ | √ |
| Prunella_strophiata |  |  |  |  |  |  |  |  |  |  |  |  |  |  | √ |  |  |  |  |  |  |  |  |  |  |  |  |  |  |  |  |  |  |  |  |  |  |
| Garrulax_berthemyi |  |  |  |  | √ |  |  | √ | √ | √ | √ |  |  |  | √ |  | √ |  |  |  |  |  | √ |  |  |  |  |  |  | √ | √ |  | √ |  |  |  |  |
