# Supplementary material for: The diversity of resident passerine bird in the East Yunnan‐Kweichow Plateau is closely related to plant species richness, vertical altitude difference and habitat area
Source: Ecol Evol. 2023 Jan 17;13(1):e9735. doi: 10.1002/ece3.9735 (PMC9843479; doi:10.1002/ece3.9735)
Supplement: Supplementary file 5 — Appendix S5. [file ECE3-13-e9735-s003.docx]

**Appendix S5 Functional traits used to measure avian community functional diversity and structure**

| **Functional trait** | **Type** | **Unit** | **Subdivision** | **Description** | **Data Sources** |
| --- | --- | --- | --- | --- | --- |
| Body mass | morphological | g |  | Body mass is related to the amount and size of food required by an individual, and dispersal distance; | Wang, Y.Song, Y.Zhong, Y.Chen, C.Zhao, Y.Di ZengWu, Y., &Ding, P., 2021. A dataset on the life-history and ecological traits of Chinese birds. Biodiversity Science, 29, 1149-1153. 10.17520/biods.2021201. |
| Wing length | morphological | mm |  | Wing length is the distance from the wrist joint (where the wing is bent) to the tip of the longest primary feather when the wings are closed; it is related to the flight ability of birds; |  |
| Culmen | morphological | mm |  | The culmen length is the distance from the tip of the mouth along the top ridge of the mouth to the base of the mouth; it is related to the feeding method and food characteristics of birds; |  |
| Tarsus length | morphological | mm |  | The tarsus length is the part from the lower part of the bird's leg to the toe; it is related to the flexibility of the bird's feet to move; |  |
| Clutch size | life history |  |  | The clutch size is the number of full clutches produced by female birds in a certain breeding season; it reflects the reproductive ability of birds; |  |
| Generation length | life history | y |  | Generation length is the normal lifespan of birds, which is related to bird competition; | International, B. (2022). Birdlife International (Data Zone). http://datazone.birdlife.org/home  IUCN. (2022). The IUCN Red List of Threatened Species. https://www.iucnredlist.org |
| Diet | life history |  | Fruits | Feed on plant fruit. | Zhao, Z. (2001). A handbook of birds of China (Volume II : Passerines)) Jilin: Jilin Science and Technology Press. |
|  |  |  | seeds | Feed on seed. |  |
|  |  |  | Plant_organs | Feed on plant twigs, leaves, flowers and buds. |  |
|  |  |  | Vertebrate | Feeds on vertebrates such as small rodents, amphibians and reptiles. |  |
|  |  |  | Insect | Feed on insects. |  |
|  |  |  | Other_invertebrates | Feed on invertebrates other than insects. |  |
|  |  |  | Nectar | Feed on nectar. |  |
|  |  |  | Carrion | Feed on carrion. |  |
| Foraging stratum | life history |  | stra_Water | Birds feeding on water, such as swallowtail, river urchins. |  |
|  |  |  | stra_Ground | Birds feeding on the ground. |  |
|  |  |  | stra_Understory | Birds foraging under vegetation. |  |
|  |  |  | stra_Midstory | Birds foraging in the middle of vegetation. |  |
|  |  |  | stra_Canopy | Birds foraging in the vegetation canopy. |  |
|  |  |  | stra_Air | Birds feeding in the air. |  |
| Nest location | life history |  | nl_Cliff_cave | Birds that nest in cliffs or caves. |  |
|  |  |  | nl_Ground | Birds that nest on the ground. |  |
|  |  |  | nl_Hollow | Birds nesting in tree holes. |  |
|  |  |  | nl_Building | Birds nesting on buildings. |  |
|  |  |  | nl_Tree | Birds nesting on foliage of plants. |  |
|  |  |  | nl_Grass | Birds nesting in the grass. |  |
