# Supplementary material for: The diversity of resident passerine bird in the East Yunnan‐Kweichow Plateau is closely related to plant species richness, vertical altitude difference and habitat area
Source: Ecol Evol. 2023 Jan 17;13(1):e9735. doi: 10.1002/ece3.9735 (PMC9843479; doi:10.1002/ece3.9735)
Supplement: Supplementary file 6 — Appendix S6. [file ECE3-13-e9735-s012.docx]

**Appendix S6** **Explanatory variables of all 37 study sites; Area, the area of the site(****the unit is km²); Plant.ric, vascular plant species richness; MAT, mean annual temperature(the unit is degrees Celsius /℃); MAP, mean annual precipitation(the unit is millimetres /mm); ASH, annual sunshine time(the unit is hour/h); AT, accumulated temperature(the unit is degrees Celsius /℃); Altitude.min, the lowest altitude(the unit is meter/m); Altitude.max, the highest altitude(the unit is meter/m); Altitude.mean, the mean altitude(the unit is meter/m); Altitude.HD, the vertical altitude difference(he unit is meter/m).**

| **Site** | **Longitude** | **Latitude** | **Area** | **Plant.ric** | **MAT** | **MAP** | **ASH** | **AT** | **Altitude.min** | **Altitude.max** | **Altitude.mean** | **Altitude.HD** |
| --- | --- | --- | --- | --- | --- | --- | --- | --- | --- | --- | --- | --- |
| AHH | 106.6477 | 26.5382 | 12.18 | 557 | 15.3 | 1129.5 | 1354 | 5583 | 1076 | 1310 | 1193 | 234 |
| BDS | 104.6295 | 25.81 | 260 | 1523 | 13.07 | 1300 | 1578.5 | 3657.46 | 760 | 2558.2 | 1769.82 | 1798.2 |
| BS | 108.7832 | 25.8249 | 28.6099 | 906 | 17 | 1305.91 | 1196.79 | 5068.22 | 650 | 1274.5 | 962.25 | 624.5 |
| BLDJ | 105.9188 | 27.2375 | 125.8 | 739 | 11.8 | 1180 | 1335.5 | 4200 | 1060 | 2121.1 | 1590.55 | 1061.1 |
| BMS | 107.431 | 27.5205 | 191.73 | 819 | 14.9 | 1310 | 1354 | 5439 | 476.4 | 1501.4 | 988.9 | 1025 |
| BQ | 107.25 | 28.66665 | 520 | 645 | 14.6 | 1382.1 | 1091.6 | 3293.27 | 700 | 2200 | 1427.82 | 1500 |
| CH | 104.2578 | 26.83665 | 96 | 672 | 10.5 | 950.9 | 1805.4 | 2568.7 | 2170 | 2400 | 2285 | 230 |
| DSH | 107.5546 | 29.16665 | 269.9 | 2836 | 12.12 | 1076 | 1108.33 | 4883 | 560 | 1900 | 1230 | 1340 |
| FJS | 108.7868 | 27.9278 | 419 | 2588 | 8.4 | 2073.7 | 1035 | 3500 | 500 | 2572 | 1277.82 | 1872 |
| FDS | 108.0667 | 27.33335 | 152 | 1616 | 14.54 | 1131.98 | 1197.6 | 4312.03 | 500 | 1869 | 978.04 | 1369 |
| FY | 107.2 | 28.63335 | 242.005 | 706 | 16.14 | 1105 | 1089 | 3449.5 | 850 | 1837 | 1343.5 | 987.8 |
| GT | 105.4061 | 26.71815 | 225 | 1243 | 12.56 | 1230 | 1350 | 3573.2 | 1052 | 2331 | 1716.59 | 1279 |
| HSH | 107.0399 | 28.0092 | 75.0055 | 506 | 14 | 1100 | 1114 | 4646.4 | 824 | 1571 | 1197.5 | 747 |
| KLXY | 107.8876 | 26.5238 | 1.1167 | 496 | 15.3 | 1285.6 | 1288 | 5218.3 | 675 | 794 | 734.5 | 119 |
| KKS | 107.1378 | 28.21525 | 262.31 | 1369 | 13.42 | 1062.8 | 1121.2 | 3843 | 650 | 1762 | 1206 | 1112 |
| LSC | 107.3584 | 26.46665 | 93.07 | 651 | 13.9 | 1465 | 1068.1 | 4471 | 1062 | 1862 | 1462 | 800 |
| LGS | 108.2417 | 26.39165 | 477.92 | 1962 | 15.5 | 1351 | 1225 | 4600 | 650 | 2178.8 | 1414.4 | 1528.8 |
| LSH | 105.9931 | 27.48845 | 100.2541 | 1001 | 14 | 1136 | 1036.95 | 4619 | 748 | 1749.8 | 1248.9 | 1001.8 |
| LY | 105.0454 | 26.0953 | 56.307 | 664 | 14.2 | 1353.8 | 1527.06 | 4050 | 700 | 2080 | 1390 | 1380 |
| MYH | 108.1977 | 28.7653 | 311.13 | 1298 | 17.6 | 1148.9 | 1105.6 | 4500 | 290 | 1441 | 865.5 | 1151 |
| ML | 107.9819 | 25.2514 | 212.85 | 977 | 15.3 | 1752.5 | 1272.8 | 4598.6 | 430 | 1078 | 754 | 648 |
| MH | 104.7853 | 26.5942 | 1.977 | 240 | 12.3 | 1182.8 | 1404.5 | 3900 | 1400 | 1900 | 1650 | 500 |
| NGO | 108.3583 | 26.49165 | 221.04 | 681 | 15 | 1096.7 | 1100 | 4250 | 555 | 1741 | 1148 | 1186 |
| NGA | 107.9661 | 28.27 | 220.04 | 1732 | 13.6 | 1200 | 940 | 4244.6 | 468 | 1445 | 956.5 | 977 |
| PG | 105.0217 | 25.0208 | 50.3 | 1216 | 15.32 | 1472.1 | 1587.8 | 5317.15 | 760 | 1553 | 1310 | 793 |
| QNSY | 107.5152 | 26.2958 | 0.9013 | 105 | 16.1 | 1431.1 | 1454.7 | 6477 | 800 | 1200 | 1000 | 400 |
| SLHT | 106.678 | 26.44509 | 2.19 | 181 | 14.9 | 1450.8 | 1162.6 | 4194.8 | 1000 | 1200 | 1100 | 200 |
| SYT | 108.0274 | 27.94806 | 174 | 984 | 14.95 | 1195.48 | 1144.55 | 5013.4 | 400 | 1000 | 700 | 600 |
| ST | 106.3154 | 25.17115 | 300 | 1609 | 19.27 | 1257.4 | 1445.92 | 6341.99 | 500 | 1507 | 1003.5 | 1007 |
| SL | 106.0418 | 28.4082 | 133 | 1775 | 18.2 | 1250 | 718.5 | 4417.78 | 303.9 | 1730.1 | 1017 | 1426.2 |
| TPS | 109.05 | 26.125 | 315.4629 | 1178 | 15.6 | 1321.9 | 1317.9 | 5360.2 | 137 | 1589 | 785 | 1452 |
| WFS | 107.8111 | 27.70565 | 89.8479 | 789 | 15.3 | 1205.6 | 1139 | 5548 | 401.45 | 1378.5 | 720 | 977.09 |
| XS | 106.2903 | 28.4736 | 519.11 | 1664 | 13.59 | 1029.53 | 1174.53 | 4112.12 | 420 | 1756 | 1120.89 | 1336 |
| YX | 108.5232 | 27.78595 | 218.7148 | 1087 | 14.53 | 1208.08 | 1202.65 | 5281.15 | 530 | 1599.9 | 796.9 | 1069.9 |
| YZ | 104.8897 | 26.2254 | 26.74 | 551 | 13 | 1239.5 | 1482.4 | 4503 | 780 | 1680 | 1230 | 900 |
| YLS | 108.2327 | 25.68375 | 328.49 | 1335 | 18.1 | 1211 | 1163 | 5445 | 310 | 1508 | 850 | 1198 |
| YTS | 108.1167 | 27.2 | 47 | 527 | 16.4 | 1117 | 1195.4 | 5585 | 600 | 1066 | 526 | 466 |
