# Supplementary material for: The diversity of resident passerine bird in the East Yunnan‐Kweichow Plateau is closely related to plant species richness, vertical altitude difference and habitat area
Source: Ecol Evol. 2023 Jan 17;13(1):e9735. doi: 10.1002/ece3.9735 (PMC9843479; doi:10.1002/ece3.9735)
Supplement: Supplementary file 7 — Appendix S7. [file ECE3-13-e9735-s011.docx]

**Appendix S7 The phylogenetic signal of each of the 26 Passeriformes resident bird functional traits in 37 study sites**

| **trait** | | **type** | **K** | **P** | **D** | **Pval1** | **Pval0** |
| --- | --- | --- | --- | --- | --- | --- | --- |
| Body_mass | | Continuous | 1.031 | 0.001 | — | — | — |
| Wing_length | | Continuous | 1.293 | 0.001 | — | — | — |
| Culmen | | Continuous | 1.065 | 0.001 | — | — | — |
| Tarsus_length | | Continuous | 1.069 | 0.001 | — | — | — |
| Clutch_size | | Continuous | 0.496 | 0.001 | — | — | — |
| Generation_length | | Continuous | 0.797 | 0.001 | — | — | — |
| diet | Fruits | Binary | — | — | -0.219 | 0.000 | 0.782 |
|  | Seeds | Binary | — | — | -0.147 | 0.000 | 0.703 |
|  | Plant_organs | Binary | — | — | 0.678 | 0.086 | 0.059 |
|  | Vertebrate | Binary | — | — | 0.110 | 0.053 | 0.536 |
|  | Insect | Binary | — | — | -0.500 | 0.000 | 0.868 |
|  | Other_invertebrates | Binary | — | — | 0.922 | 0.335 | 0.043 |
|  | Nectar | Binary | — | — | -1.396 | 0.000 | 0.934 |
|  | Carrion | Binary | — | — | 3.579 | 0.810 | 0.122 |
| foraging stratum | Stra_Water | Binary | — | — | -1.259 | 0.000 | 0.982 |
|  | Stra_Ground | Binary | — | — | 0.011 | 0.000 | 0.526 |
|  | Stra_Understory | Binary | — | — | 0.030 | 0.000 | 0.488 |
|  | Stra_Midstory | Binary | — | — | 0.354 | 0.000 | 0.100 |
|  | Stra_Canopy | Binary | — | — | -0.135 | 0.000 | 0.709 |
|  | Stra_Air | Binary | — | — | 0.037 | 0.000 | 0.477 |
| nest location | Nl_Cliff_cave | Binary | — | — | -0.470 | 0.000 | 0.927 |
|  | Nl_Ground | Binary | — | — | 0.523 | 0.012 | 0.071 |
|  | Nl_Hollow | Binary | — | — | -0.289 | 0.000 | 0.834 |
|  | Nl_Building | Binary | — | — | -4.769 | 0.000 | 1.000 |
|  | Nl_Tree | Binary | — | — | -0.239 | 0.000 | 0.826 |
|  | Nl_Grass | Binary | — | — | -0.054 | 0.000 | 0.598 |

Pval1：Probability of E(D) resulting from no (random) phylogenetic structure;

Pval0：Probability of E(D) resulting from Brownian phylogenetic structure
