# Supplementary material for: The diversity of resident passerine bird in the East Yunnan‐Kweichow Plateau is closely related to plant species richness, vertical altitude difference and habitat area
Source: Ecol Evol. 2023 Jan 17;13(1):e9735. doi: 10.1002/ece3.9735 (PMC9843479; doi:10.1002/ece3.9735)
Supplement: Supplementary file 8 — Appendix S8. [file ECE3-13-e9735-s007.docx]

**Appendix S8 Bird Diversity and Community** **phylogenetic/Functional Structure indices in 37 study sites**

| **Site** | **SR** | **PD** | **MPD** | **SESmpd** | **FD** | **MFD** | **SESmfd** |
| --- | --- | --- | --- | --- | --- | --- | --- |
| AHH | 55 | 1186.956 | 81.78894 | 0.250651 | 3.306555 | 0.231915 | 1.531168 |
| BDS | 75 | 1521.069 | 80.99582 | -0.11975 | 4.041467 | 0.229537 | 1.463153 |
| BS | 37 | 886.0277 | 80.25945 | -0.32131 | 2.332749 | 0.224322 | 0.414571 |
| BLDJ | 44 | 912.8263 | 81.52074 | 0.129744 | 2.834717 | 0.240472 | 2.280263 |
| BMS | 55 | 1146.593 | 80.26282 | -0.43379 | 3.245381 | 0.235079 | 2.019182 |
| BQ | 76 | 1423.671 | 78.17401 | -1.91702 | 3.977286 | 0.229069 | 1.443521 |
| CH | 64 | 1246.603 | 82.1563 | 0.51887 | 3.510717 | 0.23888 | 2.609216 |
| DSH | 90 | 1624.864 | 78.43098 | -2.02138 | 4.452703 | 0.22906 | 1.637534 |
| FJS | 91 | 1683.465 | 79.81175 | -1.091 | 4.278172 | 0.223628 | 0.620588 |
| FDS | 61 | 1236.698 | 81.05534 | -0.05883 | 3.297564 | 0.231732 | 1.681774 |
| FY | 56 | 1136.097 | 79.58617 | -0.7416 | 3.304651 | 0.234672 | 1.965489 |
| GT | 58 | 1176.869 | 79.57417 | -0.84294 | 3.340374 | 0.22919 | 1.154991 |
| HSH | 41 | 930.4532 | 84.10228 | 1.151467 | 2.824656 | 0.248308 | 2.925496 |
| KLXY | 27 | 703.8632 | 81.1482 | -0.0226 | 1.967762 | 0.220569 | 0.046862 |
| KKS | 86 | 1536.413 | 77.73527 | -2.54145 | 4.190642 | 0.223896 | 0.633731 |
| LSC | 54 | 1206.11 | 83.04598 | 0.859476 | 3.483234 | 0.244072 | 3.163233 |
| LGS | 79 | 1543.005 | 81.36559 | 0.086123 | 3.828724 | 0.225804 | 0.909025 |
| LSH | 55 | 1169.312 | 81.67316 | 0.232525 | 3.585779 | 0.243218 | 3.058792 |
| LY | 72 | 1392.233 | 80.53509 | -0.45857 | 3.89131 | 0.232394 | 1.93187 |
| MYH | 79 | 1486.315 | 80.13369 | -0.70242 | 4.19119 | 0.23375 | 2.36511 |
| ML | 87 | 1729.519 | 82.45562 | 0.950192 | 4.170504 | 0.221441 | 0.132402 |
| MH | 59 | 1266.134 | 83.37438 | 1.081784 | 3.527702 | 0.24442 | 3.245365 |
| NGO | 55 | 1227.84 | 80.05755 | -0.53269 | 3.273762 | 0.231599 | 1.529655 |
| NGA | 51 | 1018.713 | 81.84011 | 0.275615 | 3.238563 | 0.242522 | 2.866307 |
| PG | 74 | 1493.378 | 81.95074 | 0.489959 | 4.199548 | 0.233219 | 2.073834 |
| QNSY | 37 | 872.9575 | 81.40809 | 0.044508 | 2.608488 | 0.232501 | 1.226822 |
| SLHT | 24 | 656.7066 | 81.52491 | 0.068454 | 1.777635 | 0.228289 | 0.632733 |
| SYT | 50 | 1070.174 | 79.52235 | -0.75007 | 3.033374 | 0.232075 | 1.506164 |
| ST | 75 | 1512.227 | 82.15051 | 0.602154 | 3.932649 | 0.23153 | 2.001248 |
| SL | 85 | 1589.208 | 81.26984 | 0.039291 | 4.308331 | 0.2321 | 2.117581 |
| TPS | 55 | 1230.824 | 79.77672 | -0.63481 | 3.259996 | 0.233635 | 1.836958 |
| WFS | 41 | 900.5785 | 80.3289 | -0.32972 | 2.671548 | 0.234098 | 1.569295 |
| XS | 79 | 1428.384 | 78.46682 | -1.78446 | 4.082191 | 0.230616 | 1.773467 |
| YX | 58 | 1212.262 | 82.46183 | 0.609269 | 3.464227 | 0.239533 | 2.672172 |
| YZ | 74 | 1510.371 | 81.44359 | 0.129416 | 4.281758 | 0.236281 | 2.562055 |
| YLS | 71 | 1466.068 | 81.57905 | 0.244256 | 3.868939 | 0.233302 | 2.058869 |
| YTS | 47 | 1012.746 | 78.82931 | -0.93549 | 2.79924 | 0.229108 | 1.072554 |
