# Supplementary material for: The diversity of resident passerine bird in the East Yunnan‐Kweichow Plateau is closely related to plant species richness, vertical altitude difference and habitat area
Source: Ecol Evol. 2023 Jan 17;13(1):e9735. doi: 10.1002/ece3.9735 (PMC9843479; doi:10.1002/ece3.9735)
Supplement: Supplementary file 11 — Appendix S11. [file ECE3-13-e9735-s001.docx]

**Appendix S11** **Pearson’ s correlations among explanatory variables**

|  | **Latitude** | **Area** | **Plant.ric** | **MAT** | **MAP** | **ASH** | **AT** | **Altitude.min** | **Altitude.max** | **Altitude.mean** | **Altitude.HD** |
| --- | --- | --- | --- | --- | --- | --- | --- | --- | --- | --- | --- |
| **Longitude** | 0.21 | 0.30 | 0.21 | 0.30 | 0.22 | -0.56* | 0.38 | **-0.63*** | -0.42 | **-0.71**** | 0.09 |
| **Latitude** |  | 0.33 | 0.29 | -0.22 | -0.32 | **-0.61**** | -0.32 | -0.16 | 0.13 | -0.07 | 0.25 |
| **Area** |  |  | **0.63**** | -0.05 | 0.12 | -0.27 | -0.26 | -0.41 | 0.43 | 0.03 | **0.73**** |
| **Plant.ric** |  |  |  | -0.16 | 0.16 | -0.31 | -0.10 | -0.42 | 0.38 | -0.01 | **0.69**** |
| **MAT** |  |  |  |  | -0.16 | -0.20 | **0.62*** | -0.48 | **-0.60*** | -0.59* | -0.15 |
| **MAP** |  |  |  |  |  | -0.04 | 0.03 | -0.20 | 0.07 | -0.10 | 0.18 |
| **ASH** |  |  |  |  |  |  | 0.02 | 0.53 | 0.17 | 0.45 | -0.26 |
| **AT** |  |  |  |  |  |  |  | -0.46 | **-0.70**** | **-0.69**** | -0.29 |
| **Altitude.min** |  |  |  |  |  |  |  |  | 0.35 | **0.79**** | -0.47 |
| **Altitude.max** |  |  |  |  |  |  |  |  |  | **0.80**** | **0.66**** |
| **Altitude.mean** |  |  |  |  |  |  |  |  |  |  | 0.13 |

Absolute values larger than 0.6 were in bold. *p＜0.05, **p＜0.01.
