# Supplementary material for: The diversity of resident passerine bird in the East Yunnan‐Kweichow Plateau is closely related to plant species richness, vertical altitude difference and habitat area
Source: Ecol Evol. 2023 Jan 17;13(1):e9735. doi: 10.1002/ece3.9735 (PMC9843479; doi:10.1002/ece3.9735)
Supplement: Supplementary file 12 — Appendix S12. [file ECE3-13-e9735-s008.docx]

**Appendix S12 Pearson’s correlations among explanatory variables and diversity indices**

|  | Longitude | Latitude | Area | Plant.ric | MAT | MAP | ASH | AT | Altitude.min | Altitude.max | Altitude.mean | Altitude.HD |
| --- | --- | --- | --- | --- | --- | --- | --- | --- | --- | --- | --- | --- |
| SR | -0.15 | 0.14 | **0.61*** | **0.68**** | -0.14 | 0.18 | -0.04 | -0.29 | -0.22 | 0.49 | 0.21 | **0.63**** |
| PD | -0.14 | 0.00 | **0.57*** | **0.63**** | -0.08 | 0.23 | 0.01 | -0.23 | -0.22 | 0.46 | 0.19 | **0.61**** |
| MPD | -0.25 | -0.46 | -0.47 | -0.31 | 0.04 | 0.12 | 0.23 | 0.16 | 0.35 | -0.10 | 0.16 | -0.38 |
| SESmpd | -0.21 | -0.54 | -0.51 | -0.37 | 0.15 | 0.12 | 0.26 | 0.23 | 0.31 | -0.18 | 0.08 | -0.41 |
| FD | -0.25 | 0.13 | 0.53 | **0.60*** | -0.14 | 0.11 | -0.01 | -0.26 | -0.18 | 0.50 | 0.25 | **0.61**** |
| MFD | -0.30 | 0.16 | -0.27 | -0.28 | -0.14 | -0.37 | 0.01 | -0.04 | 0.32 | 0.13 | 0.26 | -0.12 |
| SESmfd | -0.37 | 0.18 | -0.12 | -0.11 | -0.10 | -0.39 | 0.02 | -0.08 | 0.24 | 0.23 | 0.29 | 0.04 |

*p＜0.05, **p＜0.01.
