# Supplementary material for: The diversity of resident passerine bird in the East Yunnan‐Kweichow Plateau is closely related to plant species richness, vertical altitude difference and habitat area
Source: Ecol Evol. 2023 Jan 17;13(1):e9735. doi: 10.1002/ece3.9735 (PMC9843479; doi:10.1002/ece3.9735)
Supplement: Supplementary file 13 — Appendix S13. [file ECE3-13-e9735-s005.docx]

**Appendix S13** **Pearson’s correlations among diversity indices**

|  | PD | MPD | SESmpd | FD | MFD | SESmfd |
| --- | --- | --- | --- | --- | --- | --- |
| SR | **0.98**** | -0.26 | -0.32 | **0.97**** | -0.25 | 0.02 |
| PD |  | -0.16 | -0.21 | **0.97**** | -0.23 | 0.03 |
| MPD |  |  | **0.97**** | -0.16 | **0.55*** | 0.46 |
| SESmpd |  |  |  | -0.23 | 0.50 | 0.41 |
| FD |  |  |  |  | -0.05 | 0.22 |
| MFD |  |  |  |  |  | **0.94**** |

Absolute values larger than 0.6 were in bold. * p＜0.05, **p＜0.01.
